# Arginase 1 promotes retinal neurovascular protection from ischemia through suppression of macrophage inflammatory responses

**DOI:** 10.1038/s41419-018-1051-6

**Published:** 2018-09-25

**Authors:** Abdelrahman Y. Fouda, Zhimin Xu, Esraa Shosha, Tahira Lemtalsi, Jijun Chen, Haroldo A. Toque, Rebekah Tritz, Xuezhi Cui, Brian K. Stansfield, Yuqing Huo, Paulo C. Rodriguez, Sylvia B. Smith, R. William Caldwell, S. Priya Narayanan, Ruth B. Caldwell

**Affiliations:** 1 Charlie Norwood VA Medical Center, Augusta, GA USA; 2 Vascular Biology Center, Augusta University, Augusta, GA USA; 3 James and Jean Culver Vision Discovery Institute, Augusta University, Augusta, GA USA; 4 Department of Pharmacology and Toxicology, Augusta University, Augusta, GA USA; 5 Department of Cell Biology and Anatomy, Augusta University, Augusta, GA USA; 6 Department of Pediatrics, Augusta University, Augusta, GA USA; 7 Moffitt Cancer Center, Tampa, FL USA; 8 Department of Ophthalmology, Augusta University, Augusta, GA USA; 9 Program in Clinical and Experimental Therapeutics, College of Pharmacy, University of Georgia, Augusta, GA USA

## Abstract

The lack of effective therapies to limit neurovascular injury in ischemic retinopathy is a major clinical problem. This study aimed to examine the role of ureohydrolase enzyme, arginase 1 (A1), in retinal ischemia-reperfusion (IR) injury. A1 competes with nitric oxide synthase (NOS) for their common substrate l-arginine. A1-mediated l-arginine depletion reduces nitric oxide (NO) formation by NOS leading to vascular dysfunction when endothelial NOS is involved but prevents inflammatory injury when inducible NOS is involved. Studies were performed using wild-type (WT) mice, global A1^+/^^−^ knockout (KO), endothelial-specific A1 KO, and myeloid-specific A1 KO mice subjected to retinal IR injury. Global as well as myeloid-specific A1 KO mice showed worsened IR-induced neuronal loss and retinal thinning. Deletion of A1 in endothelial cells had no effect, while treatment with PEGylated (PEG) A1 improved neuronal survival in WT mice. In addition, A1^+/−^ KO mice showed worsened vascular injury manifested by increased acellular capillaries. Western blotting analysis of retinal tissue showed increased inflammatory and necroptotic markers with A1 deletion. In vitro experiments showed that macrophages lacking A1 exhibit increased inflammatory response upon LPS stimulation. PEG-A1 treatment dampened this inflammatory response and decreased the LPS-induced metabolic reprogramming. Moreover, intravitreal injection of A1 KO macrophages or systemic macrophage depletion with clodronate liposomes increased neuronal loss after IR injury. These results demonstrate that A1 reduces IR injury-induced retinal neurovascular degeneration via dampening macrophage inflammatory responses. Increasing A1 offers a novel strategy for limiting neurovascular injury and promoting macrophage-mediated repair.

## Introduction

Ischemia-induced retinal neurovascular injury is a primary contributor in blinding diseases that affect neonates (retinopathy of prematurity), working age adults (diabetic retinopathy), and the elderly (branch vein occlusion). The retinal ischemia-reperfusion (IR) injury model has been widely used to study the mechanisms of neurovascular injury in these and other diseases of the central nervous system (CNS) such as stroke^1–5^. Therefore, it provides an excellent model to study the neurovascular damage characteristic of many CNS disorders. The lack of understanding of the mechanisms of IR injury-induced neuronal and vascular injury is a critical barrier for developing clinically effective treatments for these conditions.

Arginase has two isoforms, arginase 1 (A1) and arginase 2 (A2)^6^. A1, the cytosolic isoform, is strongly expressed in the liver, where it is the central player in the urea cycle^7^. The mitochondrial isoform, A2, is expressed in extrahepatic tissues, especially the kidney^8^. Both isoforms are expressed in the retina and brain^9^, and have been linked to CNS diseases^10^. A1 is expressed in retinal glia^10^. After experimental stroke, A1 has been reported to be strongest in myeloid cells with less expression in astrocytes^11,12^. A1 and nitric oxide synthase (NOS) enzyme compete for their common substrate the semi-essential amino acid l-arginine^13^. A1 upregulation can lead to suppression of nitric oxide (NO) formation by endothelial NOS (eNOS) resulting in superoxide production, endothelial dysfunction, platelet aggregation, and leukocyte activation and attachment to the vessel wall^14^. However, A1 expression in “M2-like” anti-inflammatory myeloid cells is thought to reduce NO production by iNOS, and thus can dampen oxidative stress and inflammation^15,16^. Interestingly, the number of A1^+^, Iba1^+^ macrophages/microglia is correlated with post-stroke neuron survival and recovery in mice^11^. Recent studies have shown that A1 is expressed exclusively by infiltrating myeloid cells and not by microglia after CNS injury^17,18^.

We have previously shown that A2 plays a deleterious role in retinal IR injury^19^. Moreover, retinal IR injury is associated with increased expression of A2 and iNOS, and decreased A1^19^. While A1 is a marker for M2 macrophages and is known to improve tissue repair, its role in macrophage polarization and neurovascular damage after CNS IR injury has not been studied^10^. Here we examined for the first time the role of A1 in retinal IR injury using mice with global and cell-specific A1 deletion. We also tested the therapeutic potential of PEGylated A1 (PEG-A1, a drug form of A1 that is currently under investigation as cancer therapy^20–24^) in retinal IR injury.

## Results

### A1 deletion worsens IR-induced neurovascular degeneration in vivo

**Fig. 1:**
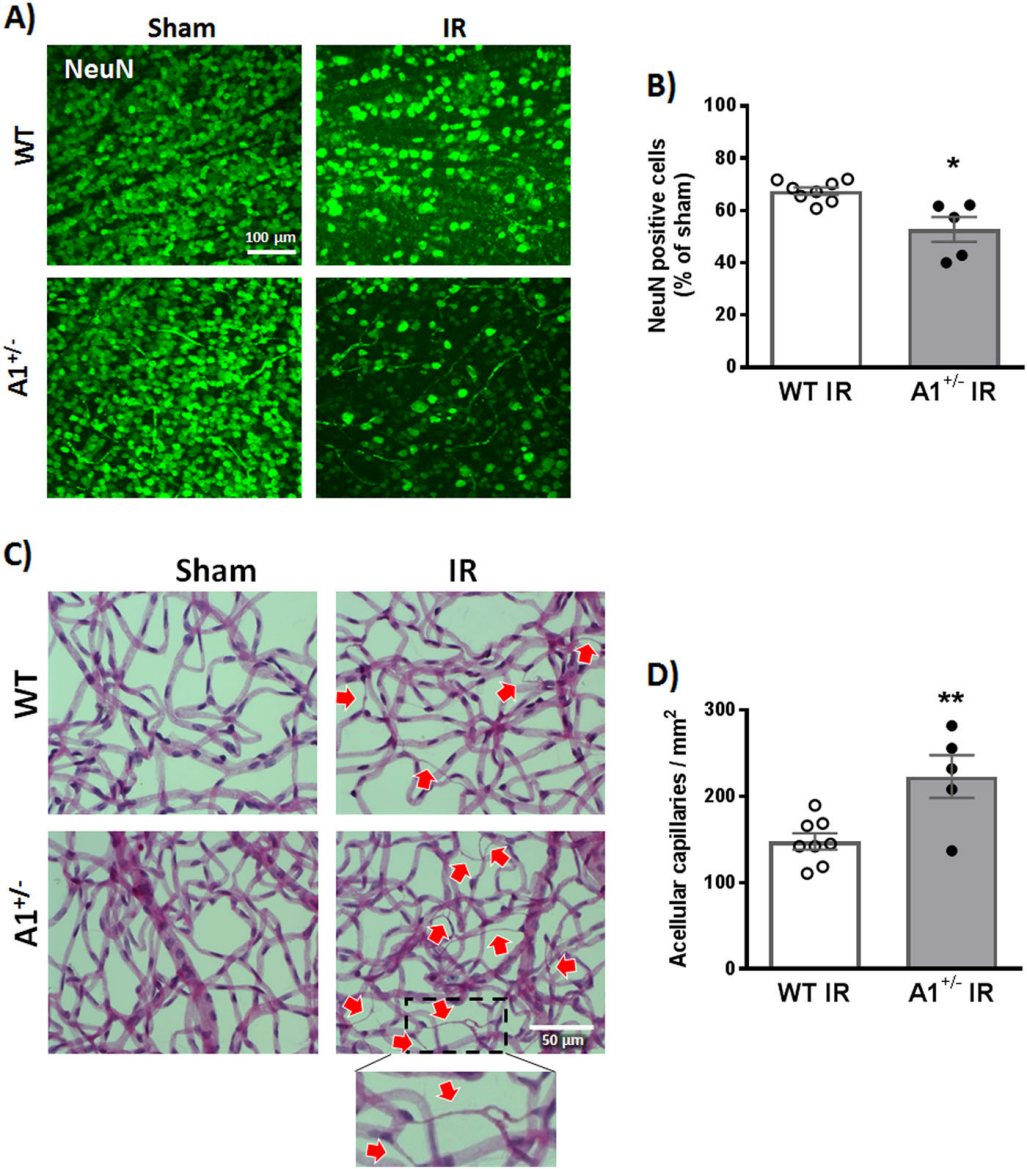
A1 deletion worsens neuronal and microvascular degeneration after IR injury. **a** WT and A1^+/−^ mice were subjected to retinal IR injury and sacrificed at 7 days. Flat-mount NeuN staining showed neuronal cell loss in WT retinas after IR injury compared to shams, which was further aggravated in A1^+/−^ mice. Scale bar = 100 μm. **b** Quantification of NeuN-positive cells, *n* = 5 for WT IR and 8 for A1^+/−^ IR, *p < 0.05. **c** Vascular digests at 14 days showed increased numbers of acellular capillaries (red arrows) in WT IR injured retinas and this microvascular degeneration was further augmented in A1^+/−^ IR injured retinas. Scale bar = 50 μm. **d** Quantification of acellular capillaries (empty basement membrane sleeves—enlarged in inset), *n* = 5 for WT IR and 8 for A1^+/−^ IR, **p < 0.01

We have previously shown that retinal IR injury is associated with decreased A1 mRNA at 3 h^19^. In line with this, we found a sustained decrease in retinal arginase activity starting at 3 h after IR injury and up to 48 h (Fig. S1). To study the role of A1 in retinal IR injury, we used heterozygous (A1^+/^^−^) global KO mice, since homozygous deletion of A1 is postnatal lethal^25^. WT or A1^+/−^ KO mice were subjected to 40 min of ischemia on the right eye followed by reperfusion as explained in the methods^26^. The left eye served as sham control. The retinal IR injury model is associated with both neuronal and microvascular degeneration that are manifested by neuronal loss and acellular capillary formation^19^. To evaluate neurodegeneration after IR injury, we labeled WT and A1^+/−^ KO retinas with the neuronal marker, NeuN and imaged the surviving neurons in the retinal ganglion cell layer by confocal microscopy^19,26^. IR injury reduced NeuN-positive cells in WT mice at 7 days, which was further worsened in A1^+/−^ mice (Fig. 1a, b). We next examined microvascular degeneration by preparing retina vascular digests and counting the number of acellular capillaries^19,27^. WT IR injured retinas showed a large number of acellular capillaries (≈150/mm^2^ empty basement membrane sleeves, red arrows, Fig. 1c, d) at 14 days after IR injury and this was further increased by ≈50% in A1^+/−^ mice.

### A1 deletion exacerbates retinal thinning and distortion after IR injury

**Fig. 2:**
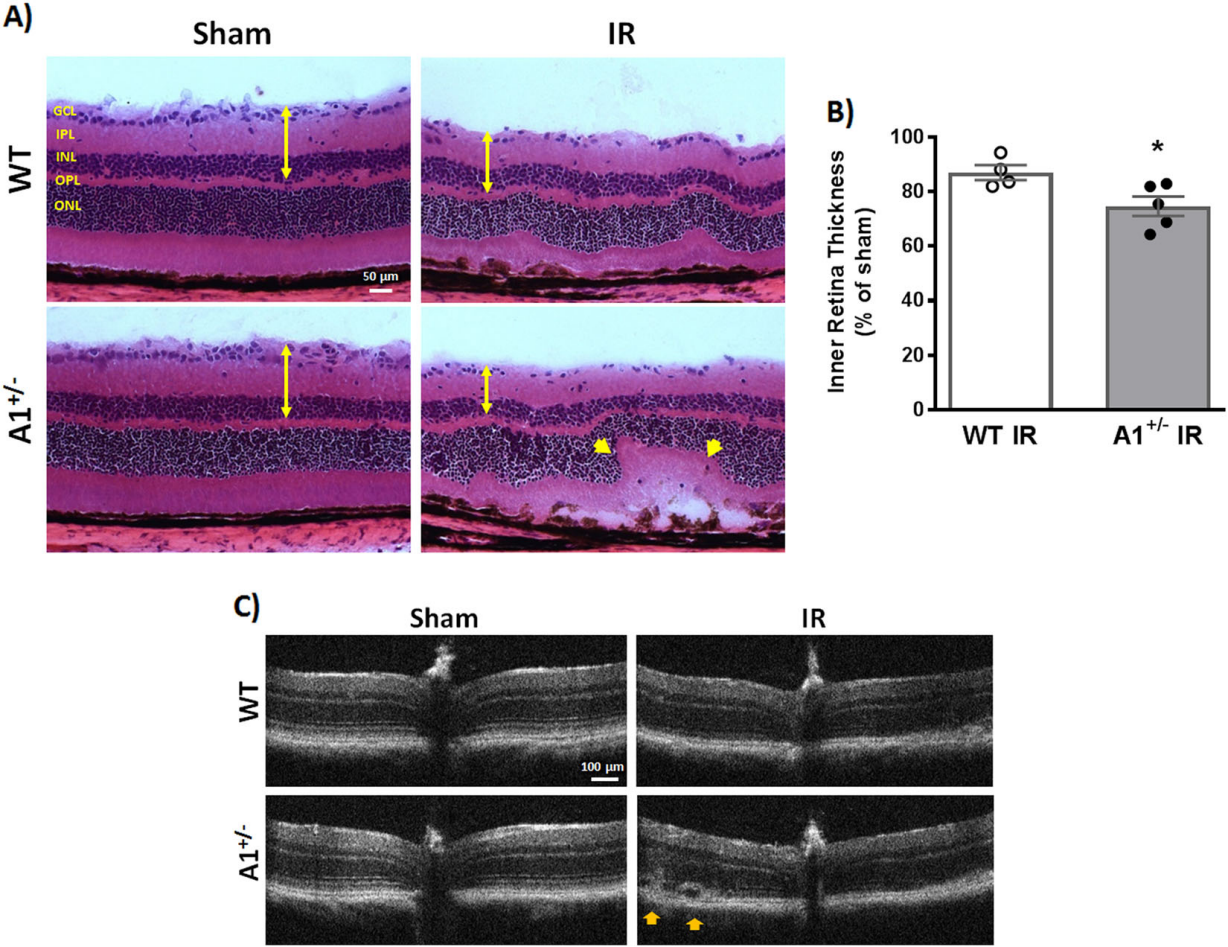
A1 deletion worsens retinal thinning and distortion after IR injury. **a** Hematoxylin and eosin (H&E) staining of retinal frozen sections showed less retinal ganglion cells, distorted morphology, and retinal thinning 7 days after IR injury which was further worsened in A1^+/−^ retinas (yellow arrow heads). Scale bar = 50 μm. GCL ganglion cell layer, IPL inner plexiform layer, INL inner nuclear layer, OPL outer plexiform layer, ONL outer nuclear layer. **b** Quantification of inner retina thickness (GCL + IPL + INL, denoted by yellow arrows in panel (**a**)), *n* = 4 for WT IR and 5 for A1^+/−^ IR, *p < 0.05. **c** Optical coherence tomography (OCT) in live mice at 7 days corroborated the H&E results with yellow arrow heads pointing at retinal distortion/detachment, *n* = 3 per group (different cohort of mice than the one used for H&E)

The IR injury model has been shown to affect the inner retinal layers (ganglion cell layer (GCL), inner plexiform layer (IPL), and inner nuclear layer (INL)) to a greater extent than the outer retina leading to reduced inner retina thickness^26,28,29^. In accordance with this, morphometric analysis on hematoxylin and eosin (H&E)-stained WT IR injured retina sections at 7 days showed reduced thickness of the inner retinal layers compared to sham controls. A1^+/−^ retinas showed further thinning and distortion compared to WT after IR injury (Fig. 2a, b). This was confirmed by optical coherence tomography (OCT) that showed worsened retinal detachment in A1^+/−^ retinas (Fig. 2c).

### A1 deletion exacerbates retinal inflammation and necroptosis after IR injury

Next, we examined the underlying mechanism of increased retinal cell death in A1^+/−^ mice after IR injury. Various mechanisms of retinal cell death have been described in the retina IR injury model with studies from our lab and others emphasizing a prominent role of programmed cell death by necroptosis (a caspase-independent programmed form of cell death)^19,30–35^. Necroptosis is associated with an early increase in cell membrane permeability. We evaluated this through propidium iodide (PI) uptake, which is plasma membrane impermeable and only labels the DNA of dying cells. We observed PI-positive cells in GCL and INL of WT retinas within 6 h following IR injury with more cells in A1^+/−^ retinas (Fig. S2).

**Fig. 3:**
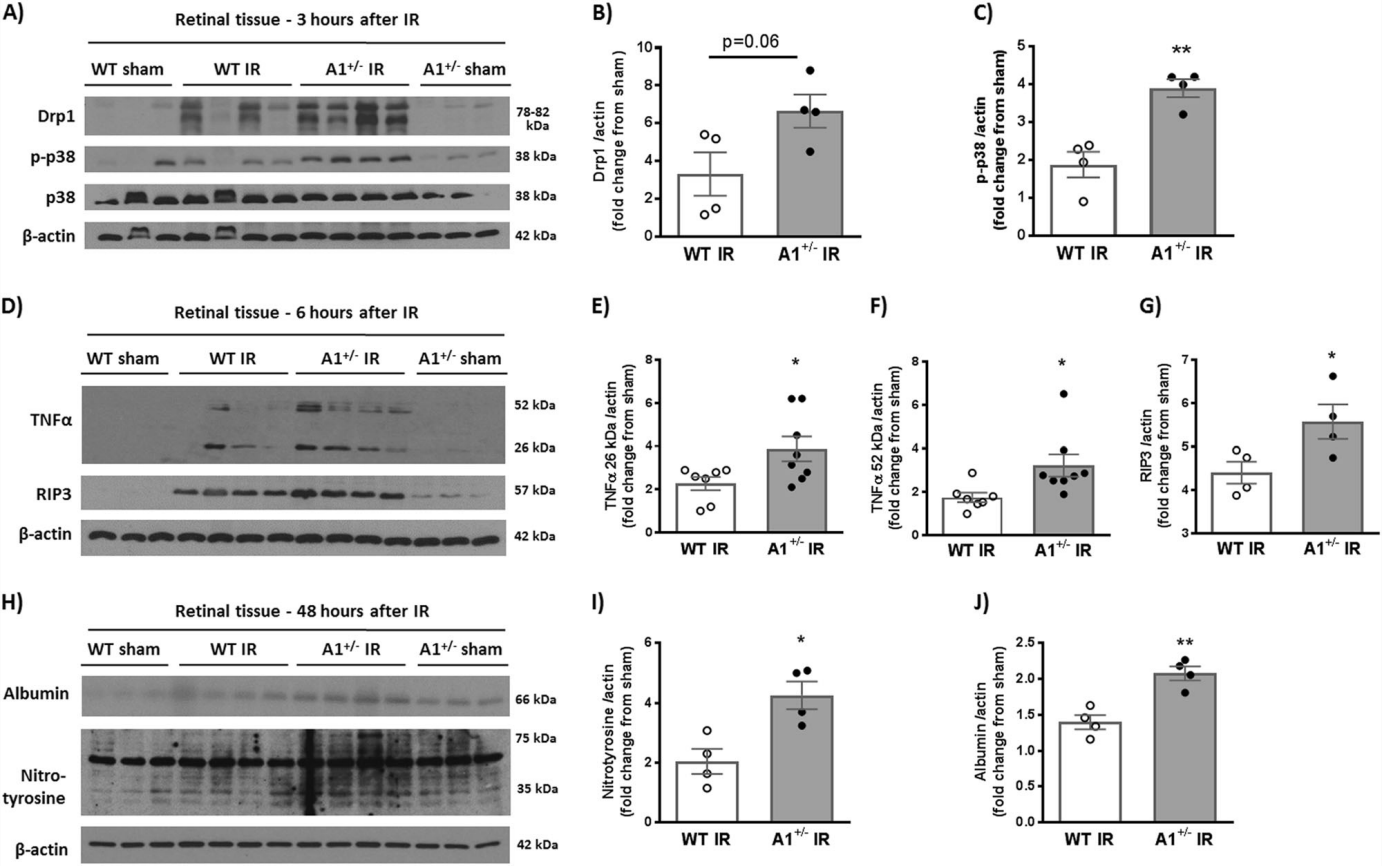
A1 deletion increases inflammation, oxidative stress, and necroptosis markers after IR injury. **a** Western blotting on retinal tissues collected at 3 h after IR showed higher levels of the stress marker p-p38 in A1^+/−^ mice compared to WT after IR injury. There was also a trend towards higher levels of the mitochondrial fission protein, Drp1. **b**, **c** show quantification of Drp1 and p-p38 respectively. **d** Analysis at 6 h after IR injury showed a similar trend with increased TNF-α (26 kDa, membrane bound and 52 kDa, homotrimeric form), and RIP3 in A1^+/−^ retinas as compared to WT. **e**−**g** show quantification of TNF-α bands and RIP3 respectively. **h** A1^+/−^ mice showed increased nitrotyrosine (marker for peroxynitrite-mediated oxidative stress via protein nitration) and albumin extravasation (measure of permeability) at 48 h after IR injury. **i**, **j** show quantification of nitrotyrosine and albumin western blotting respectively. *p < 0.05, **p < 0.01

Unlike apoptosis, necroptosis is associated with release of cellular contents and subsequent inflammatory response. Therefore, we examined the necroptosis marker receptor interacting protein 3 kinase (RIP3) together with other inflammatory markers via western blotting. Western blot analyses showed increases in the stress marker phospho-p38 MAPK in A1^+/^^−^ retinas at 3 h after IR injury as compared to WT retinas. There was also a trend towards an increase in the mitochondrial fission marker, dynamin-related protein (Drp1) but the difference was not statistically significant (Fig. 3a−c). Furthermore, IR injury induced increases in the inflammatory cytokine, tumor necrosis factor alpha (TNF-α), and RIP3 in A1^+/^^−^ retinas at 6 h (Fig. 3d−g). The increase in inflammation was associated with increases in oxidative stress. This was shown by increased nitrotyrosine formation (a measure of protein tyrosine nitration via peroxynitrite, which is formed by the reaction of NO with superoxide anion). Albumin extravasation (measure of vascular permeability) was also increased in A1^+/^^−^ as compared to WT mice at 48 h after IR injury (Fig. 3h−j).

### Effect of cell-specific A1 deletion on neuronal survival and retinal tissue thinning after IR injury

**Fig. 4:**
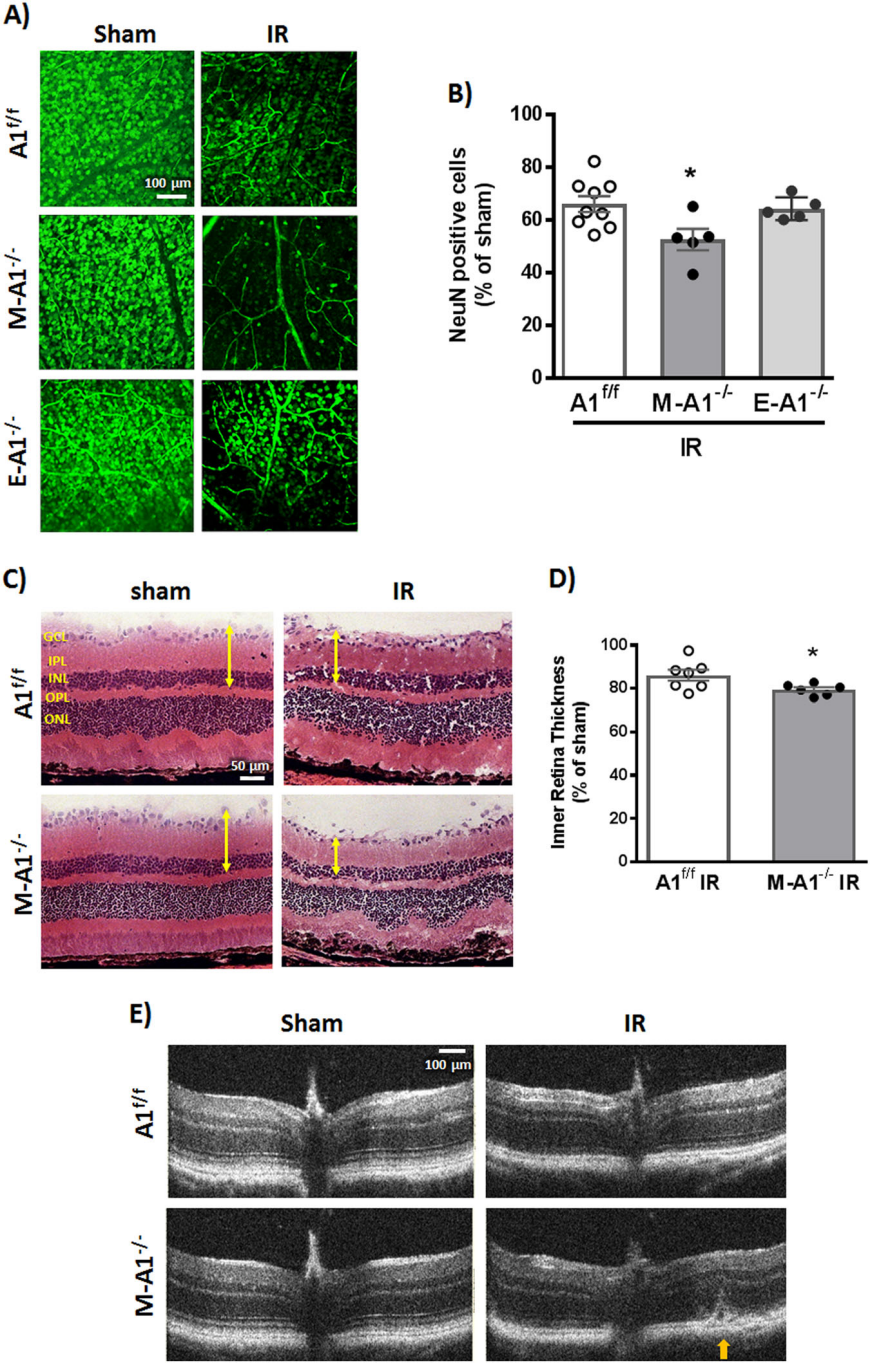
Myeloid A1 deletion worsens neuronal loss and retinal thinning after IR injury. **a** Retinas of mice with myeloid but not endothelial-specific A1 deletion showed worsened neuron loss compared to floxed control at 7 days after IR injury. Scale bar = 100 μm. **b** Quantification of NeuN-positive cells, *n* = 9 for A1^f/f^ IR and 5 for M-A1^−/−^ and E-A1^−/−^ IR, *p < 0.05 vs. A1^f/f^. **c** H&E staining at 7 days showed worsened inner retina thinning in M-A1^−/−^ mice compared to control (A1^f/f^). Scale bar = 50 μm. **d** Quantification of inner retina thickness, *n* = 7 for A1^f/f^ IR and 6 for M-A1^−/−^ IR, *p < 0.05. **e** Optical coherence tomography (OCT) corroborated the H&E results with yellow arrow pointing at retinal detachment

It has been shown that retinal IR injury induces macrophage/microglia recruitment and proliferation within 24 h with a peak in cell number at 3–5 days^36^. In accordance, we have seen an increase in Iba1-positive cells in the retina after IR injury (Fig. S3A). Interestingly, we detected Iba1-positive cells in the vitreous at 48 h after IR injury, suggesting infiltration of systemic monocyte-derived macrophages (Fig. S3B). Building on this and since global A1 deletion showed a worsened retinal IR injury outcome, we next examined the cell-specific role of A1. For this, A1 floxed (loxP) mice were crossed to LysM^cre^ and Cdh5^cre^ transgenic mice to generate mice lacking A1 in myeloid (LysM^cre^;A1^f/f^) or endothelial (Cdh5^cre^;A1^f/f^) cells, respectively. Mice with myeloid but not endothelial A1 deletion showed exacerbated neuronal loss at 7 days after IR injury compared to littermate floxed control (Fig. 4a, b). Moreover, myeloid A1 KO retinas showed more thinning and distortion after IR injury (Fig. 4c, d). Western blotting on retinal lysates from endothelial-specific A1 KO mice showed no difference in albumin extravasation at 48 h after IR injury as compared to floxed controls (Fig. S5). Collectively, these data suggest a major protective role of myeloid A1 and a minimal role of endothelial A1 in retinal IR injury. In line with a reparative role of infiltrating macrophages in the retinal IR injury model, systemic macrophage depletion using clodronate liposomes led to worsened neurodegeneration and retinal hemorrhage after IR injury in WT mice (Fig. S7).

### PEGylated A1 treatment protected retinal neurons and increased microglia/macrophages after IR injury

**Fig. 5:**
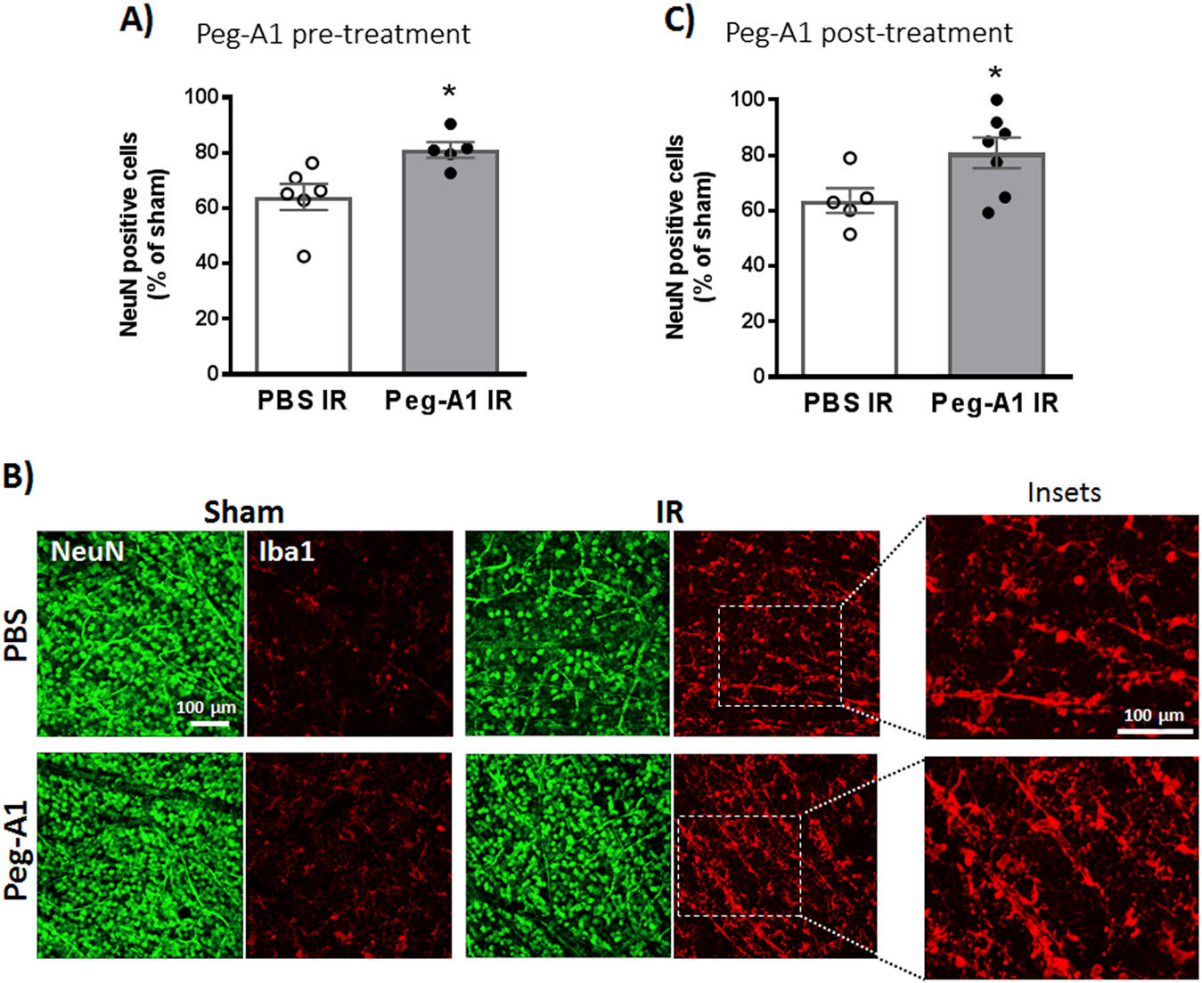
A1 treatment protects retinal neurons, and increases microglia/ macrophages after IR injury. **a** Mice received intravitreal injection of PEG-A1 (1.7 ng/µl) 3 h before induction of IR injury and were sacrificed at day 7. PEG-A1 treatment preserved retinal neurons (NeuN-positive cells), *n* = 6 for PBS and 5 for PEG-A1, *p < 0.05. **b** PEG-A1-treated retinas showed increased microglia/macrophage infiltration as evident by Iba1 staining on retina flat-mounts. **c** Treatment with PEG-A1 (1.7 ng/µl) 3 h after IR injury achieved similar neuronal preservation to the pretreatment, thus showing post-injury protective effect, *n* = 5 for PBS and 7 for PEG-A1, *p < 0.05

To study the effect of increasing A1 levels on neurodegeneration, we used PEG-A1, which is an investigational drug with good safety, pharmacodynamic, and pharmacokinetic profiles in patients^20^. WT mice received intravitreal injection of PEG-A1 (1.7 ng in 1 μl), 3 h before or after IR injury. Either pre- or post-treatment with PEG-A1 improved neuronal survival at 7 days after IR injury. Interestingly, the PEG-A1-mediated neuronal preservation was associated with more retinal macrophages/microglia with elongated morphology as evident by Iba1 staining of retina flat-mounts (Fig. 5a−c).

### A1 deletion augments macrophage inflammatory response in vitro and PEGylated A1 treatment mitigates it

To further confirm our in vivo data, we tested the role of A1 expression in macrophage inflammatory response in vitro. Peritoneal macrophages isolated from myeloid A1 KO and floxed littermate controls were treated with interleukin-4 (IL-4, 20 ng/ml) or lipopolysaccharide (LPS, 100 ng/ml) for 24 h to generate anti-inflammatory (M-2 like) or proinflammatory (M-1 like) responses, respectively. As expected, IL-4 treatment increased A1 expression in macrophages isolated from control mice.

**Fig. 6:**
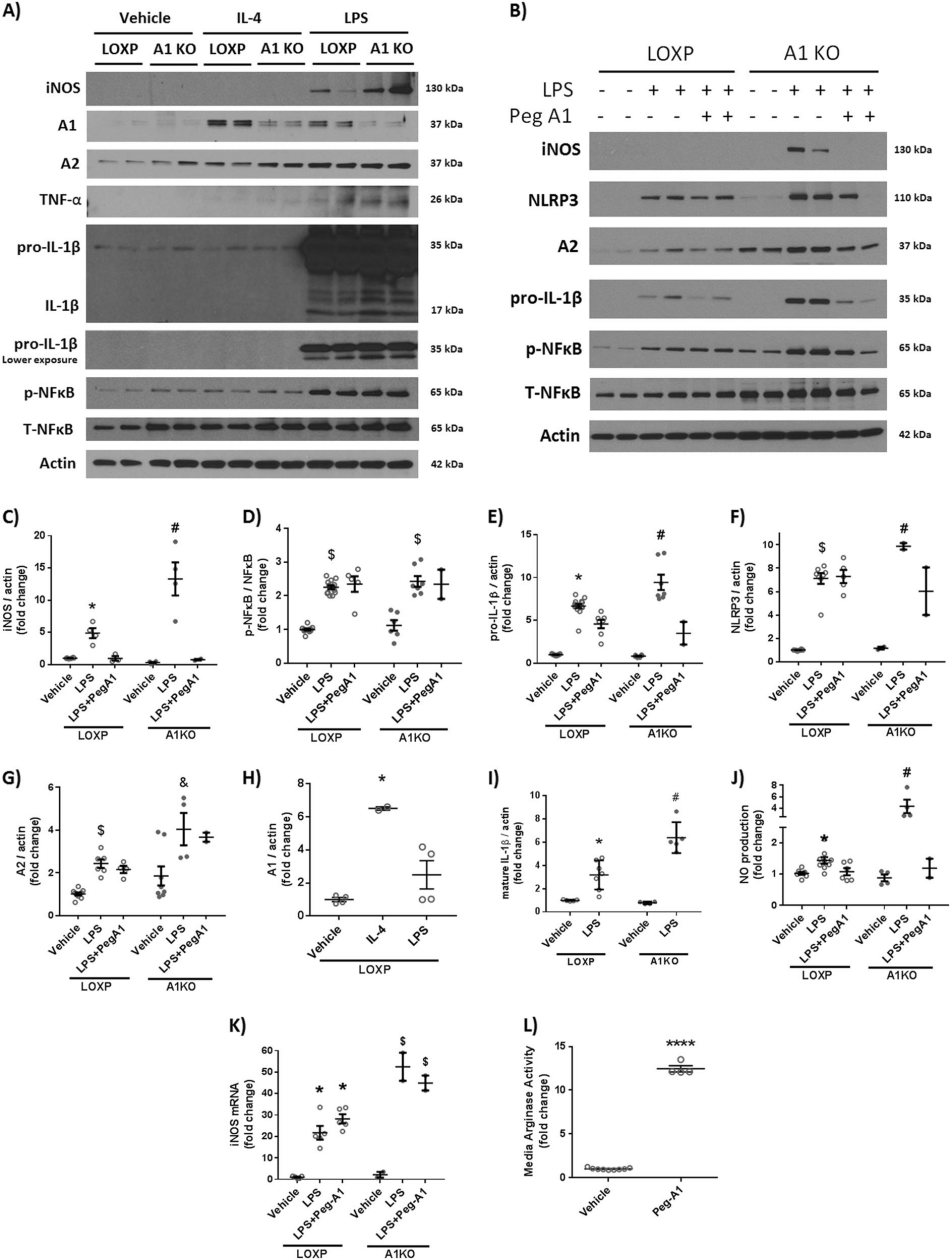
Macrophages lacking A1 show a more pronounced inflammatory response to LPS stimulation in vitro and PEG-A1 treatment mitigates it. **a** Western blotting of peritoneal macrophage cell lysates showed increased iNOS expression, TNF-α, and pro-IL-1β upon LPS stimulation which was further augmented in A1 KO macrophages. **b** PEG-A1 treatment (1 μg/ml) reduced this inflammatory response. **c**−**i** Quantification of western blot bands. *p < 0.05 vs. loxP vehicle and loxP LPS + PEG-A1, ^#^*p* < 0.05 vs. loxP LPS, A1KO vehicle and A1KO LPS + PEG-A1, ^$^*p* < 0.05 vs. respective vehicle. ^&^*p* < 0.05 vs. loxP LPS. **j** A1 KO macrophages showed more nitric oxide (NO) release into the media in response to LPS, as measured using NO analyzer and this was ameliorated by PEG-A1, *p < 0.05 vs. vehicle loxP, ^#^*p* < 0.05 vs. loxP LPS, A1KO vehicle, A1KO LPS + PEG-A1. **k** RT-PCR on BMDMs showed increased iNOS mRNA expression with LPS that was further increased in A1 KO macrophages. PEG-A1 treatment did not affect iNOS mRNA expression *p < 0.05 vs. loxP vehicle, ^$^*p* < 0.05 vs. respective loxP group. **l** Media from wells treated with PEG-A1 show marked elevation of arginase activity (12-fold increase compared to control) at the end of a 24 h incubation, ****p < 0.0001

Upon LPS stimulation, macrophages lacking A1 showed more iNOS expression, TNF-α and inflammasome pathway activation (NLRP3, NFkB, and pro-IL1β) compared to controls. Furthermore, they showed increased NO production in cell supernatant compared to controls as measured by NO analyzer. Collectively, A1 KO macrophages exhibited a more pronounced inflammatory response to LPS stimulation. Cotreatment with PEG-A1 (1 μg/ml) dampened the LPS-induced inflammatory response and reduced NO production in both control and A1 KO macrophages (Fig. 6).

### PEGylated A1 rescues LPS-induced mitochondrial dysfunction in macrophages

**Fig. 7:**
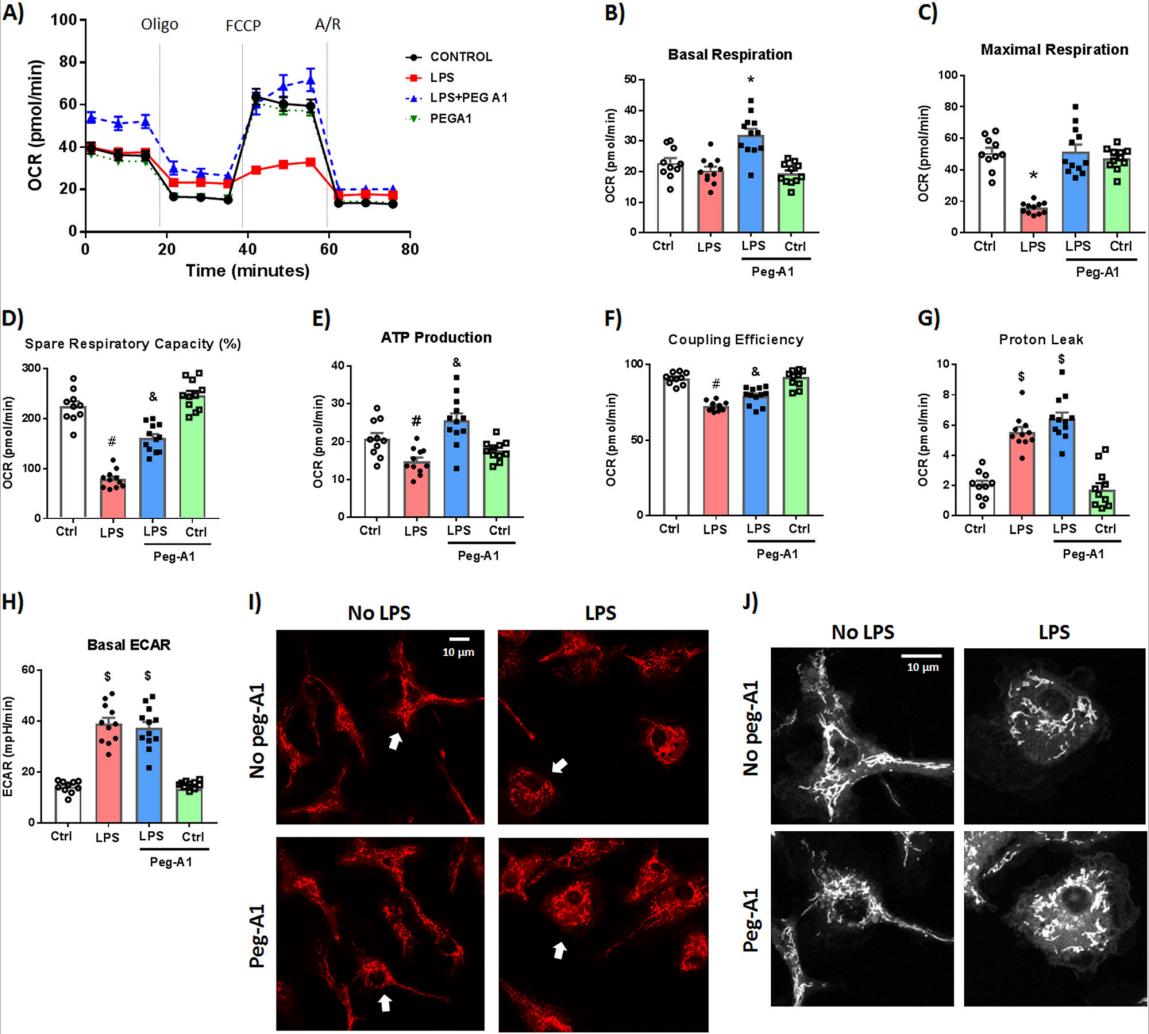
PEG-A1 treatment protects against LPS-induced mitochondrial dysfunction in WT bone marrow-derived macrophages (BMDMs). WT BMDMs were stimulated with LPS (100 ng/ml) for 24 h ± PEG-A1 (1 μg/ml). Seahorse XFe96 analyzer was used to evaluate mitochondrial function by measuring the oxygen consumption rate (OCR). **a** Change in OCR with time in response to Mito Stress test inhibitors (oligomycin, FCCP, and rotenone/antimycin A). **b**−**g** Mitochondrial respiration parameters were decreased with LPS treatment. PEG-A1 significantly rescued this decrease. *p < 0.01 vs. other three groups, ^$^*p* < 0.01 vs. controls, ^&^*p* < 0.01 vs. respective control, LPS, ^#^*p* < 0.01 vs. respective control, and LPS + PEG-A1, *n* = 12 per group. Representative run from two independent experiments that showed the same results. **h** Extracellular acidification rate (ECAR), a measure of glycolysis, was increased with LPS stimulation but was not affected by PEG-A1 cotreatment, ^$^*p* < 0.01 vs. controls, *n* = 12 per group. **i** WT macrophages under control condition or PEG-A1 treatment alone exhibited an elongated and interconnected mitochondria (stained with Rhodamine 123). LPS-induced mitochondrial fragmentation and localization around the nucleus consistent with a round activated macrophage morphology. PEG-A1 cotreatment of LPS-stimulated macrophages partially reversed the LPS effect. **j** Magnification of cells denoted by arrows in panel (**i**). Images were converted to black and white for clarity. Scale bar = 10 μm

Next, we examined macrophage metabolic reprogramming, which has been implicated in macrophage polarization and inflammatory response. Seahorse XFe96 analyzer was used to evaluate mitochondrial function by measuring the oxygen consumption rate (OCR). As previously described, LPS stimulation (100 ng/ml) shifted WT bone marrow-derived macrophages (BMDMs) to a more glycolytic phenotype (as measured by increased extracellular acidification rate, ECAR) and decreased mitochondrial respiration parameters (OCR)^37^. PEG-A1 (1 μg/ml) significantly inhibited the LPS-induced alterations in mitochondrial function parameters in WT BMDMs (Fig. 7a−g). In addition, staining live BMDMs with the mitochondrial membrane potential sensitive dye, Rhodamine 123^38^, showed mitochondrial fragmentation in response to LPS stimulation which was partially reversed with PEG-A1 cotreatment (Fig. 7i, j). Seahorse analysis of LPS-stimulated A1 KO macrophages showed impaired mitochondrial function as compared to loxP controls (Fig. S8). PEG-A1 treatment of A1 KO macrophages blunted the LPS-induced impaired mitochondrial function (Fig. S9).

NO has been shown to decrease mitochondrial reserve capacity in endothelial cells^39^. We aimed to further examine the link between A1 and mitochondrial respiration in endothelial cells. Bovine retinal endothelial cells were subjected to oxygen-glucose deprivation (OGD) for 5 h followed by 1 h reoxygenation (R). OGD/R impaired mitochondrial respiration parameters and PEG-A1 treatment (1 μg/ml) improved maximal respiration and spare respiratory capacity (difference between maximal respiration and basal respiration) after OGD/R (Fig. S10). Collectively, PEG-A1 treatment rescued the LPS- and OGD/R-induced mitochondrial dysfunction in macrophages and endothelial cells respectively.

### Intravitreal injection of A1 KO BMDMs is associated with worsened neurodegeneration after IR injury

**Fig. 8:**
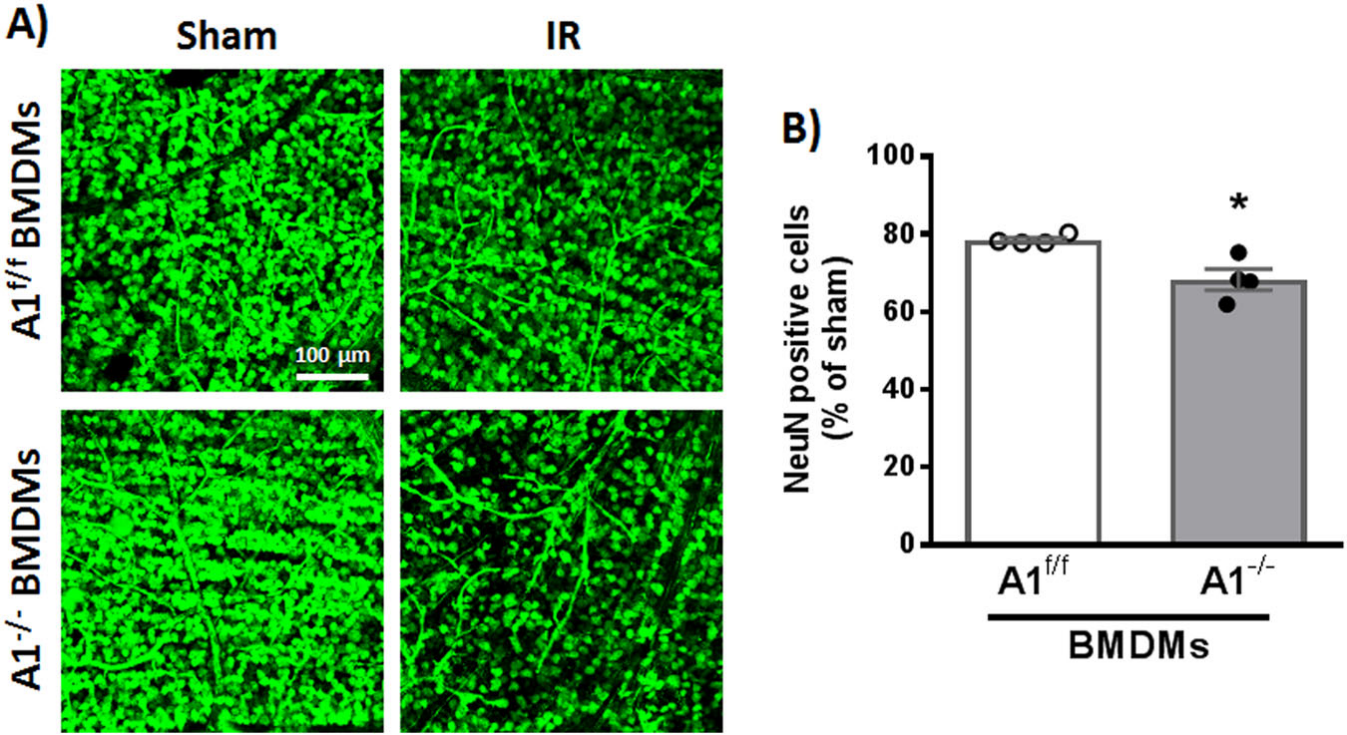
Macrophages lacking A1 worsen neurodegeneration after IR injury. **a** NeuN staining showing increased neurodegeneration in WT retinas treated with A1 KO BMDMs (2×10^5^ cells, injected intravitreally on day 3 after IR injury), **b** Quantification of NeuN-positive cells, *n* = 4 per group, *p < 0.05. Scale bar = 100 μm

Finally, we employed intravitreal injections of BMDMs to further examine the role of A1 in the macrophage reparative/damaging functions. Mice were subjected to IR injury and intravitreal macrophage injection was conducted at day 3. Retinas were collected for NeuN staining at day 7. Indeed, A1 KO macrophage injection was associated with worsened neurodegeneration after IR injury compared to loxP control macrophages (Fig. 8).

## Discussion

In this study, we present evidence on the important role of A1 in macrophage polarization toward a reparative phenotype leading to neurovascular recovery after retinal IR injury. We show that global as well as myeloid-specific A1 deletion worsens retinal IR injury outcomes and macrophages lacking A1 exhibit enhanced inflammatory response. Additionally, PEG-A1 treatment reduces retinal IR injury and dampens the macrophage inflammatory response.

Although retinopathies are diagnosed primarily based on vascular abnormalities, studies have demonstrated that inflammation^40–42^ and neurodegeneration^43,44^ occur before appearance of typical vascular pathology. Our present study in a retinal IR injury mouse model shows that A1 protects against neuronal and vascular injury. This was evident by the worsened neurodegeneration, acellular capillary formation, retinal thinning, and necroptosis in A1^+/−^ mice compared to WT. By contrast, our recent study in A2^−/−^ mice suggests a deleterious role of A2 in retinal IR injury. This could be explained by the differential expression and subcellular localization patterns of the two arginase isoforms. Both of our studies have employed different endpoints to confirm the role of A1 and A2 in retinal IR injury. Other studies have also shown opposing roles of A1 vs. A2 under different disease conditions. In fact, these two isoforms are transcribed from two different genes and appear to function independently in different tissues^10,14^. However, the possible reciprocal regulation and interaction between A1 and A2 remains to be elucidated.

Necroptosis is initiated in response to a death receptor signaling and upon failure of apoptosis induction. TNF-α binding to its receptor can induce necroptosis with RIP3 being the main executioner. Necroptosis leads to membrane permeability and release of cell components, which causes an inflammatory response. RIP3 can also directly activate the NLRP3 inflammasome pathway. We and others have shown activation of the necroptosis pathway within hours of the IR injury which can induce inflammation and myeloid cell recruitment. Our previous publication^19^, and current data show that while A2 deletion reduces IR injury-induced necroptosis in the retina, A1 deletion can augment it.

Our molecular analyses show that A1 deletion is associated with upregulation of iNOS, peroxynitrite, TNF-α, and RIP3. A1 competes with NOS enzymes for their common substrate l-arginine leading to less NO production^13^. A1 upregulation can decrease NO production by iNOS in myeloid cells, thus reducing oxidative stress and inflammation^15,16^. However, chronic A1 upregulation in endothelial cells can lead to uncoupling of endothelial NOS (eNOS), via reduction of l-arginine, resulting in endothelial dysfunction^14^. In our acute model of retinal IR injury, we did not detect any A1-mediated adverse vascular outcomes. In fact, A1^+/−^ mice showed more microvascular degeneration and greater vascular permeability which was not affected by endothelial-specific deletion of A1. Furthermore, PEG-A1 treatment rescued the OGD/R-induced mitochondrial dysfunction in endothelial cells. Collectively, this suggests a vascular protective role of A1 in our acute injury model in young and otherwise healthy mice. Possible adverse vascular effects of A1 treatment in mouse models of endothelial dysfunction will be addressed in future studies.

A1 has been long used as a marker for anti-inflammatory M2-like macrophages; however, its direct functional role has not been examined in CNS diseases. Using myeloid-specific A1 KO mice as well as in vitro macrophage experiments, we show here that this protective role of A1 in IR injury is mediated through myeloid cells. NO induces TNF-α expression in myeloid cells and potentiates its neurotoxicity^45–54^. Our results suggest that A1 decreases iNOS-mediated NO production and oxidative stress in myeloid cells leading to decreased TNF-α production and subsequent necroptosis and tissue damage. Moreover, NO has been linked to macrophage metabolic reprogramming to a more glycolytic phenotype and less mitochondrial oxidative phosphorylation through nitrosylation of the electron transport chain complexes^55^. In line with this, our data show that PEG-A1 effectively rescues LPS-induced suppression of mitochondrial metabolism. Interestingly, NFkB activation in response to LPS stimulation was not changed by A1 deletion or PEG-A1 treatment suggesting that A1 regulates iNOS and inflammatory cytokines transcription at a level downstream of NFkB phosphorylation. One possibility is that A1 suppresses inflammatory gene transcription in macrophages through epigenetic modification. A recent report has shown that, putrescine, a downstream product of arginase can suppress M1 inflammatory gene transcription through histone modification and altered euchromatin formation^56^. Further studies are needed to elucidate the mechanisms by which A1 promote a less inflammatory macrophage phenotype.

Monocyte-derived macrophages infiltrate the CNS after injury and recent reports have examined their role in CNS injury outcome. Two independent studies have shown that brain-infiltrating macrophages after ischemic stroke acquire an M2-like reparative phenotype^57,58^. Monocyte-derived macrophages have been shown to prevent hemorrhagic transformation and mediate long-term functional recovery after stroke in mice^59,60^. Moreover, brain-infiltrating macrophages reduce lesion volume and neurological deficit in an intracerebral hemorrhage (ICH) mouse model through M2 polarization^61^. Interestingly, macrophages have been shown to promote vascular repair after traumatic brain injury in mice^62^, and directly repair cerebrovascular ruptures in zebrafish^63^. On the other hand, one report showed that macrophage depletion reduced myelin damage and promoted neurological recovery in a mouse stroke model^64^. Our current data show that macrophages play a protective role in retinal IR injury and their depletion further worsens neurodegeneration and hemorrhage. Moreover, we show that A1 is a central player in this protective effect. While our in vitro studies focused on A1-mediated dampening of the macrophage inflammatory response, the in vivo studies suggest that A1 can promote a reparative macrophage phenotype as well. The proposed protective role of macrophages in our model could be mediated through clearing of dying cells via phagocytosis and promoting vascular repair. Further studies are warranted to examine these mechanisms.

In conclusion, our study shows that A1 ameliorates the IR injury-induced retinal injury via dampening the macrophage inflammatory response. Enhancing myeloid A1 can be a potential therapeutic approach for treatment of CNS ischemic conditions, especially ischemic retinopathies.

## Materials and methods

### Mouse retinal IR injury model

All animal procedures were performed in accordance with the Association for Research in Vision and Ophthalmology (ARVO) Statement and adhered to the Public Health Service Policy on Humane Care and Use of Laboratory Animals (revised July 2017). Procedures were approved by the institutional animal care and use committee (Animal Welfare Assurance no. A3307–01). Male mice (10−12 weeks old) were anaesthetized with ketamine/xylazine mixture and the right eye was subjected to IR injury while the left eye served as sham control. IR injury was induced by inserting a needle connected to an elevated saline reservoir into the anterior chamber to raise the intraocular pressure to 110 mmHg for 40 min as previously described^19,26^. The mice were sacrificed at various times after IR injury to examine different endpoints based on the literature and our previous publications^19,26^.

### Experimental groups

To determine the role of A1 in neurovascular degeneration after IR injury, we used mice with global and conditional A1 deletion: heterozygous A1^+/−^ knockout (KO), endothelial-specific (Cdh5^Cre^;A1^f/f^ or E-A1^−/−^) KO, and myeloid-specific (LysM^Cre^; A1^f/f^ or M-A1^−/−^) A1 KO. A1^+/−^ mice on C57BL6J background were used since A1^−/−^ is postnatal lethal due to hyperamonemia^25,65–67^. Experiments were conducted on littermate control and KO mice to ensure proper comparison. IR injury was conducted on all mice within a litter during the same day. Mice were selected for surgery in a random order irrespective of the genotype. Cell-specific A1 KO mice were generated by crossing C57BL-6 A1 floxed mice (A1^f/f^) with Cre-expressing transgenic mice under control of the VE-Cadherin promoter (Cdh5^Cre^) or lysozyme 2 promoter (LysM^Cre^) to generate endothelial-specific (E-A1^−/−^) or myeloid-specific (M-A1^−/−^) mice respectively. We have recently characterized and confirmed the E-A1^−/−^ mice^68^. Myeloid A1 deletion has been confirmed by tissue immunostaining and western blotting on isolated cells (Fig. S4). Further details are provided in the supplementary data.

Five sets of in vivo experiments were conducted:

*Experiment* 1: A1^+/−^ KO mice and C57BL6J WT controls were used to examine the effect of whole body deletion of one copy of A1 on IR injury outcome.

*Experiment* 2: Endothelial-specific (E-A1^−/−^) and myeloid-specific M-A1^−/−^ mice were used and compared to floxed controls (A1^f/f^) to examine the cell-specific role of A1.

*Experiment* 3: WT mice were treated with clodronate liposomes to deplete systemic monocytes/macrophages or control liposomes to examine the role of these cells in retinal IR injury.

*Experiment* 4: WT mice were treated with intravitreal injection of PEG-A1 (1.7 ng in 1 μl, dose was selected based on preliminary studies) to examine the effect of increasing intraocular arginase on retinal IR injury outcome.

*Experiment* 5: WT mice received intravitreal injection of A1^−/−^ or A1^f/f^ macrophages to examine the impact of A1 expression in macrophages on IR injury outcome.

### Monocyte/macrophage depletion

Two hundred microliters of clodronate or red fluorescent control liposomes (Encapsula Nanosciences) were injected intraperitoneally 1 day before and at day 3 after IR injury. This achieved ≈80% systemic monocyte/macrophage depletion at day 7 after IR injury (Fig. S6). Clodronate induces apoptosis when the liposomes are engulfed by macrophages while control liposomes were used to control for the induction of macrophage phagocytic activity. Cages were changed every 2-3 days to maintain a clean environment and mice were closely monitored for any signs of infections.

### PEGylated arginase 1 (PEG-A1) treatment

PEG-A1 (1.7 ng/μl) or phosphate-buffered saline (PBS) was administered under anesthesia via intravitreal injection (in 1 µl volume) using a Hamilton syringe 3 h before or after induction of retinal IR injury.

### Intravitreal macrophage injection

Cultured BMDMs were suspended in PBS and injected intravitreal (2×10^5^ per 1 µl) on day 3 after IR injury.

### Evaluation of neurodegeneration

Neuronal degeneration was assessed at 7 days after IR injury as previously described^19,26^. Eyeballs were collected and fixed overnight in 4% paraformaldehyde at 4 °C, then retinas were dissected into flat-mounts and stained for the neuronal marker, NeuN (Millipore, Cat. # MAB377, Billerica, MA). Four images were taken in the retina midperiphery using a confocal microscope (LSM 510; Carl Zeiss, Thornwood, NY) and NeuN-positive cells were counted using ImageJ software. Results are presented as a percent of NeuN-positive cell numbers in the GCL of the IR injured eyes compared to the sham eyes.

### Retinal vasculature isolation and measurement of acellular capillaries

Vasculature was isolated at 14 days after IR injury via trypsin digestion of retinas that were dissected from overnight fixed eyeballs as previously described^19,69^. The isolated retinal vasculature was air-dried on silane-coated slides and stained with periodic acid-Schiff and hematoxylin. Acellular capillaries were counted in ten random fields of the mid-retina. The number of acellular capillaries was divided by the field area to get number of acellular capillaries per 1 millimeter square (mm^2^) of retina.

### Histology and morphometric analysis

Retinal structure was assessed at 7 days on anesthetized mice using OCT (the Bioptigen Spectral Domain Ophthalmic Imaging System, SDOIS; Bioptigen Envisu R2200, NC) as previously described^70^. Retinal thickness was determined by morphometric analysis on H&E-stained retinal frozen cross sections as previously described^19,26,70^. Inner retina (INL + IPL + GCL) thickness was measured on H&E images at three different distances from optic nerve head using ImageJ software. Averaged retinal thickness was presented as percentage compared to the contralateral sham eyes.

### Western blot analysis

For in vivo experiments, retinas were collected from the mice, snap-frozen in dry ice, and stored at −80 °C. For analysis of albumin leakage across the blood-retinal barrier, mice were transcardially perfused with PBS to clear blood out of the retina vessels before collection and retinas were processed for western blotting. Retinas were homogenized using a hand homogenizer in RIPA lysis buffer and centrifuged at 20,000 × *g* to prepare the protein extracts. Protein concentration was measured using Pierce BCA protein assay kit (Thermo Scientific). For in vitro experiments, media were collected and cells were washed with ice-cold PBS then collected in RIPA buffer to prepare the cell lysates. Retinas or cell lysates were run on SDS-PAGE then transferred to nitrocellulose membranes (Millipore, Billerica, MA). The membranes were probed with following primary antibodies prepared in 2% BSA: A1 (Santa Cruz Biotechnology Cat. # Sc-20150, 1:500 dilution), phospho p38 (Cell Signaling Technology Cat. # 4511, 1:500), total p38 (Cell Signaling, Cat. # 9212, 1:500), Drp1 (Santa Cruz Biotechnology, Cat. # SC-271583, 1:500), TNF-α (Abcam, Cat. # ab1793), albumin (Bethyl Laboratories, 1:5000), RIP3 (Santa Cruz Biotechnology, Cat. # SC-135170, 1:500), β-actin (Sigma-Aldrich Cat. # A1978, 1:5000), anti-nitrotyrosine antibody (Millipore, Cat. # 05-233, 1:5000), iNOS (Cell Signaling, Cat. # 13120), A2 (Santa Cruz Biotechnology, Cat. # Sc-20151, 1:500 dilution), IL-1β (R&D, Cat. # AF-401-NA), p-NFkB (Cell Signaling, Cat. # 3033, 1:500), T-NFkB (Cell Signaling, Cat. # 4764, 1:500), NLRP3 (Cell Signaling, Cat. # 15101). Secondary antibodies (GE Healthcare) were prepared in 5% milk in 1:2000 dilution. Bands were quantified using ImageJ and normalized to β-actin loading control. For the nitrotyrosine blot, all bands in each lane were quantified except for the thick albumin band (~66 kDa) that appears due to the reaction of the secondary anti-mouse antibody with mouse albumin in the tissue extracts.

### Isolation and culture of primary macrophages

#### Peritoneal macrophages

Mice were injected intraperitoneally with 5 ml of 3% Brewer’s thioglycollate medium (Sigma) as described previously^71^. Mice were sacrificed 3-5 days later and peritoneal macrophages were collected in PBS through peritoneal lavage. Cells were centrifuged and then plated in six-well plates (1 million per well) in DMEM containing penicillin/streptomycin (P/S), and 10% fetal bovine serum (FBS). Medium was changed after 2 h to remove nonadherent nonmacrophage cells. Experiments were done on day 2 in DMEM containing 1% PS and 2% FBS.

#### Bone marrow-derived macrophages

Bone marrow cells were isolated and differentiated in vitro into macrophages based on a published protocol^72^. Briefly, both femurs and tibias were harvested and flushed with 20−25 ml sterile PBS using a 27-gauge needle. Flushed cells in PBS were spun down and re-suspended in differentiation medium (DMEM high glucose containing 20% FBS, 20% L929 conditioned media, and 1% P/S). Cells were subsequently plated on 100 mm dishes (uncoated, for easier cell detachment). Media was replaced with fresh differentiation media on day 4. On day 7, media was removed and cells were rinsed with sterile PBS twice. Cells were subsequently gently scraped and collected in PBS, spun down, re-suspended in normal growth media (DMEM high glucose containing 20% FBS, and 1% P/S), and then plated in 12-well plates for in vitro polarization or used for in vivo treatment.

##### Macrophage polarization

Cells were stimulated with LPS (100 ng/ml) or interleukin 4 (IL-4, 20 ng/ml) for 24 h to achieve proinflammatory (M1) or anti-inflammatory (M2) phenotype respectively. In some experiments, macrophages were cotreated with LPS and PEG-A1 (1 μg/ml) to examine the effect of PEG-A1 on the LPS-induced macrophage inflammatory response.

### Arginase activity assay

Arginase activity assay was conducted as previously described^9^. Briefly, the enzyme was activated by heating the lysate or supernatant at 56 °C in 25 mM Tris buffer (pH 7.4) containing 5 mM MnCl_2_. l-Arginine hydrolysis was then conducted by incubating 50 μl of the activated samples with 50 μl of 0.5 M l-arginine (pH 9.7) at 37 °C for 60 min. The reaction was stopped by adding 400 μl of acid solution mixture (H_2_SO_4_:H_3_PO_4_:H_2_O, 1:3:7). The concentration of urea, which is the end product of l-arginine hydrolysis by arginase, was determined after adding 25 μl of 9% α-isonitrosopropiophenone and heating the mixture at 100 °C for 45 min. Urea standards and 200 μl of each sample were transferred to a 96-well plate and read at 540−550 nm in a BioTek microplate reader. Protein concentration in the lysates was determined by a BCA assay (Pierce Biotechnology). Arginase activity was calculated as mmol urea/mg protein and as percent of control.

### Nitric oxide (NO) measurement

We measured nitrite (NO_2_^−^), the stable breakdown product of NO in cell-conditioned medium to reflect NO production by macrophages. The conditioned media were collected at the end of experiments and injected in glacial acetic acid containing sodium iodide. NO_2_ is quantitatively reduced to NO under these conditions, which can be quantified by a chemiluminescence detector after reaction with ozone in an NO analyzer (Sievers, Boulder, CO)^68^.

### Quantitative RT-PCR

Total RNA was extracted from macrophages using TRIzol reagent (Invitrogen, CA, USA). RNA was converted to cDNA using M-MLV reverse transcriptase (Invitrogen, CA, USA). Quantitative PCR for iNOS gene expression was performed using an ABI StepOne Plus Thermocycler (Applied Biosystems, CA, USA) with SYBR Green dye. Forward primer 5′-GTT CTC AGC CCA ACA ATA CAA GA-3′, reverse primer 5′-GTG GAC GGG TCG ATG TCA C3′. Data were normalized to HPRT and the fold change between levels of different transcripts was calculated by the ΔΔCT method.

### Seahorse XFe96 Mito stress test

Mito Stress test (Agilent, Cat. # 103015-100, Santa Clara, CA) and Seahorse XFe96 (Agilent, Santa Clara, CA) were used to evaluate mitochondrial function as previously described^37^. Briefly, Seahorse 96-well cell culture plates were used for growing the cells. At day 7, BMDMs were seeded at cell density of 40 K/well in the Seahorse cell culture plate in all the wells except A1, A12, H1 and H12 wells which were used as background wells. The plate was left under the hood for 1 h to ensure even distribution of cells, and then cells were checked under microscope and put in the incubator. Cells were maintained to grow in normal complete media (DMEM, 20% FBS, 1% P/S) for 3−5 h. Then, cells were treated with LPS overnight in DMEM media supplemented with 10% FBS. The Seahorse media was prepared according to manufacturer’s instructions and supplemented with 4 mM glutamine (Gemini, West Sacramento, CA), 1 mM pyruvate (Gemini, West Sacramento, CA), and 25 mM glucose (Sigma, St. Louis, MO). On the day of the assay, the pH of the media was adjusted to 7.4 ± 0.1, and the Mito stress test was conducted according to the manufacturer’s instructions. The concentrations of the injection compounds used were as follows: oligomycin (1 µM), FCCP (1 µM) and rotenone/antimycin A (0.5 µM). The data were collected and analyzed using the Wave software (Agilent).

### Mitochondrial staining

Vectacell^TM^ Rhodamine 123 (Vector Laboratories) was used to label mitochondria in live cells according to the manufacturer’s instructions. Briefly, cells were washed three times with modified PBS containing 1 mM CaCl_2_ and 0.5 mM MgCl_2_ (PBS^+^) (Thermo Fisher). The cells were incubated with the Rhodamine 123 staining solution in 37 °C for 15 min. Then, the cells were rinsed three times with PBS^+^. Zeiss LSM 780 Inverted Confocal microscopy (Carl Zeiss AG, Oberkochen, Germany) was used to image the live cells using a ×63 lens. Several images were taken randomly to cover the whole field.

### Statistical analysis

Statistical analysis was conducted using GraphPad Prism 7 software. Values were tested to assess whether they followed a normal distribution by the same software. One-way or two-way ANOVA with post-hoc Tukey multiple comparisons was used to analyze the statistical significance of experimental results in studies of three or more groups. The significance of differences between two groups was determined by Student’s *t*-test. *p* < 0.05 was considered significant. Sample size for each experiment was decided based on our previously published work. Outliers were checked by GraphPad online outlier calculator. For in vitro studies, each experiment was performed in triplicates and repeated with at least three different batches of isolated primary cells. Graphs were prepared using GraphPad Prism 7 software, and results were expressed as means ± standard errors of the mean (SEM).

## Disclaimer

The contents do not represent the views of the Department of Veterans Affairs or the United States Government. The funders had no role in study design, data collection and analysis, decision to publish, or preparation of the manuscript.

## Electronic supplementary material

Supplementary data
