# Supplementary material for: Arginase 1 promotes retinal neurovascular protection from ischemia through suppression of macrophage inflammatory responses
Source: Cell Death Dis. 2018 Sep 25;9(10):1001. doi: 10.1038/s41419-018-1051-6 (PMC6156564; doi:10.1038/s41419-018-1051-6)
Supplement: Supplementary file 1 — Supplementary data [file 41419_2018_1051_MOESM1_ESM.pdf]

Supplementary Data: Arginase 1 promotes retinal neurovascular protection from ischemia through suppression of macrophage inflammatory responses

Supplementary materials and methods

Propidium iodide labeling and detection of necrotic cells

Propidium iodide (PI) was used to label necrotic cells as previously described.[1] Briefly, PI (P-3566, Sigma, 5 mg/kg) was injected intraperitoneally at 5 h after IR injury and mice were sacrificed 1 h later by carbon dioxide (CO_2) inhalation followed by cervical dislocation. Eyeballs were harvested and snap-frozen in OCT compound for sectioning. Retinal sections (10 µm) were fixed in 100% ethanol for 10 min at room temperature. PI-positive cells were examined in 3 sections from each sample using a Carl Zeiss Anxioplan2 fluorescence microscope.

Immunofluorescence

Eyes were enucleated and fixed overnight in 4% PFA at 4 °C then switched to 30% sucrose overnight at 4 °C. Eye balls were then embedded in optimal cutting temperature (O.C.T) solution and cryostat sections (10 µm) were prepared. Sections were stained as previously described.[2] The following primary antibodies were incubated overnight at 4 °C: anti-Iba1 (Wako, Cat. # 019-19741, 1:400), anti-TSPO (Abcam, Cat. # ab118913, 1:200), followed by fluorescent secondary antibodies (Invitrogen, 1:500). The same fixation, sectioning and staining protocol was used for spleen and liver tissues.

If needed, image contrast enhancement was conducted on representatives from all experimental groups using imageJ software.

Cell specific A1 KO mice

Cell specific A1 KO mice were generated by crossing C57BL-6J A1 floxed mice with LoxP sites on either side of exons 7 and 8 of A1 ($A1^{f/f}$ Stock No. 008817, Jackson Labs) with transgenic mice containing Cre recombinase as follows: For endothelial specific A1 deletion, we used mice expressing Cre under control of the VE-Cadherin ($Cdh5^{Cre}$, Stock No. 017968) promoter. The $Cdh5^{Cre}$ line exhibits uniform Cre expression in endothelial cells of both developing and quiescent vessels. For myeloid specific A1 deletion, we used mice expressing Cre under control of the myeloid lineage-specific promoter of the lysozyme 2 gene (*Lyz2*) ($LysM^{Cre}$, Stock No. 004781), The $LysM^{Cre}$ line exhibits uniform Cre expression in cells of the myeloid cell lineage, including monocytes, granulocytes, mature macrophages and a small subset of microglia (about 30%). $A1^{f/f}$ mice, which have the same phenotype as C57BL/6J WT mice, were used as knockout controls. Genotype is determined by PCR using tail genomic DNA. We have previously characterized and confirmed the endothelial specific A1 KO mice by measuring A1 with PCR and western blotting in isolated endothelial cells as well as staining aortic sections with anti-CD31 and anti-A1 antibodies.[3] Myeloid specific deletion was confirmed by performing western blotting on peritoneal macrophages and staining spleen sections with anti-Iba1 (Wako, Cat. # 019-19741, 1:400) and anti-A1 (Santa Cruz Biotechnology) antibodies (fig. S4).

Endothelial cells culture and oxygen-glucose deprivation/reoxygenation (OGD/R) protocol

Bovine Retinal Endothelial Cells (BRECs) were isolated and cultured as previously described.[4] Oxygen-glucose deprivation/reoxygenation (OGD/R) in vitro was used to mimic *in vivo* ischemia/reperfusion injury. For OGD/R experiments with BRECs, cells (passages 4-6) were seeded in the Seahorse cell culture plate coated with 0.2% gelatin. Cells were maintained in

normal complete media for 24 h. The next day, cells were switched to serum-free media overnight and then subjected to 5 h of OGD (incubated in a hypoxia chamber (ProOx 110, Biospherix, <1% O_2, 94% N_2, and 5% CO_2 at 37 °C) and glucose free DMEM medium). OGD was followed by 1 h-reoxygenation (R) in complete medium under normoxic conditions (95% air, 5% CO2 at 37 °C). Durations were selected based on our preliminary experiments. Cells were treated with PEG-A1 (1 µg/ml) during OGD and at reoxygenation to examine the effect of A1 modulation on the OGD/R-induced mitochondrial dysfunction. The day before the assay, the Seahorse media was prepared according to manufacturer's instructions and supplemented with 2.5 mM glutamine (Gemini, West Sacramento, CA) and 5.5 mM glucose (Sigma, St. Louis, MO). On the day of the assay, we measured the pH of the media and kept it at 7.4±0.1. Then, we ran the Mito stress test according to manufacturer's instructions.

Supplementary figures and figure legends

S1

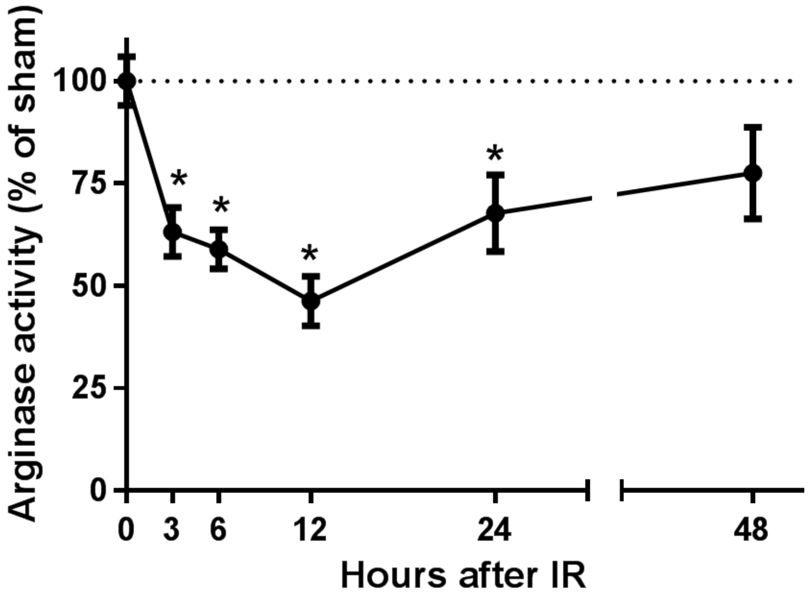

Fig S1. Decreased arginase activity in retinal tissue after IR injury. Arginase activity was determined in WT retinal tissues collected at 3, 6, 12, 24 and 48 h after IR injury and compared to shams. There was a significant decrease in arginase activity after IR injury for the first 24 h. Arginase activity declined to about 46 % of sham at 12 h and then increased thereafter, *p<0.05 vs sham, n=5 per group.

S2

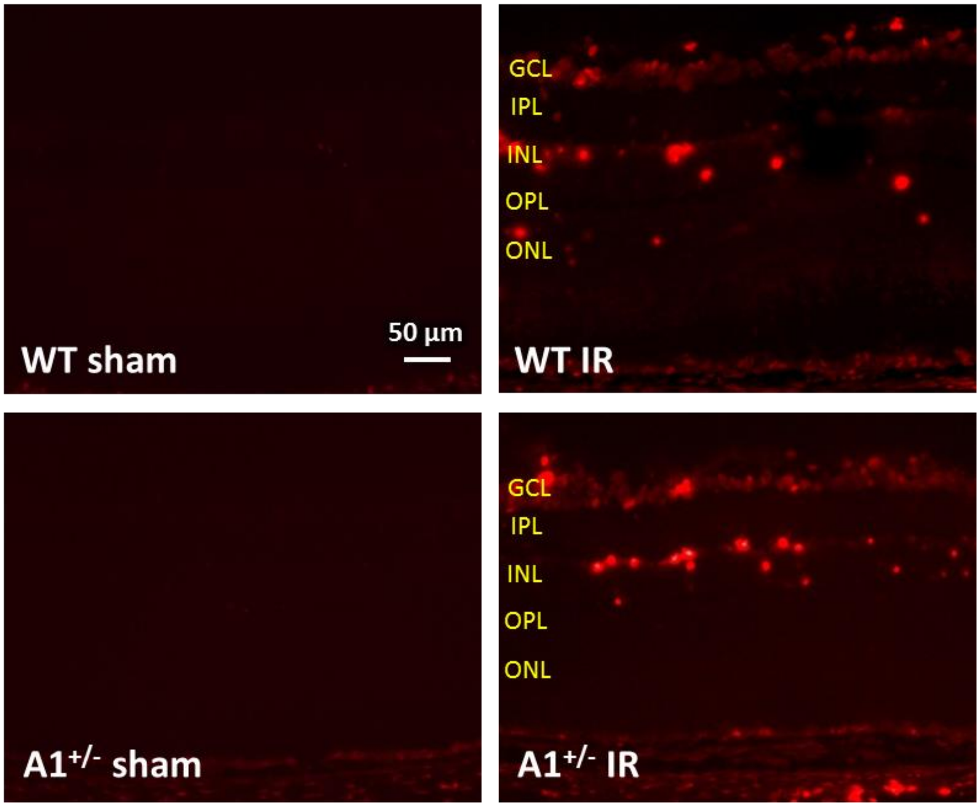

Fig S2. A1 deletion and necroptotic cell death. A) Retina cross-section images showing propidium iodide (PI) labeling of dying cells at 6 h after IR injury (PI was injected i.p. 5 mg/kg, 1 h before animal sacrifice), n=5. Scale bar = 50 μm.

S3

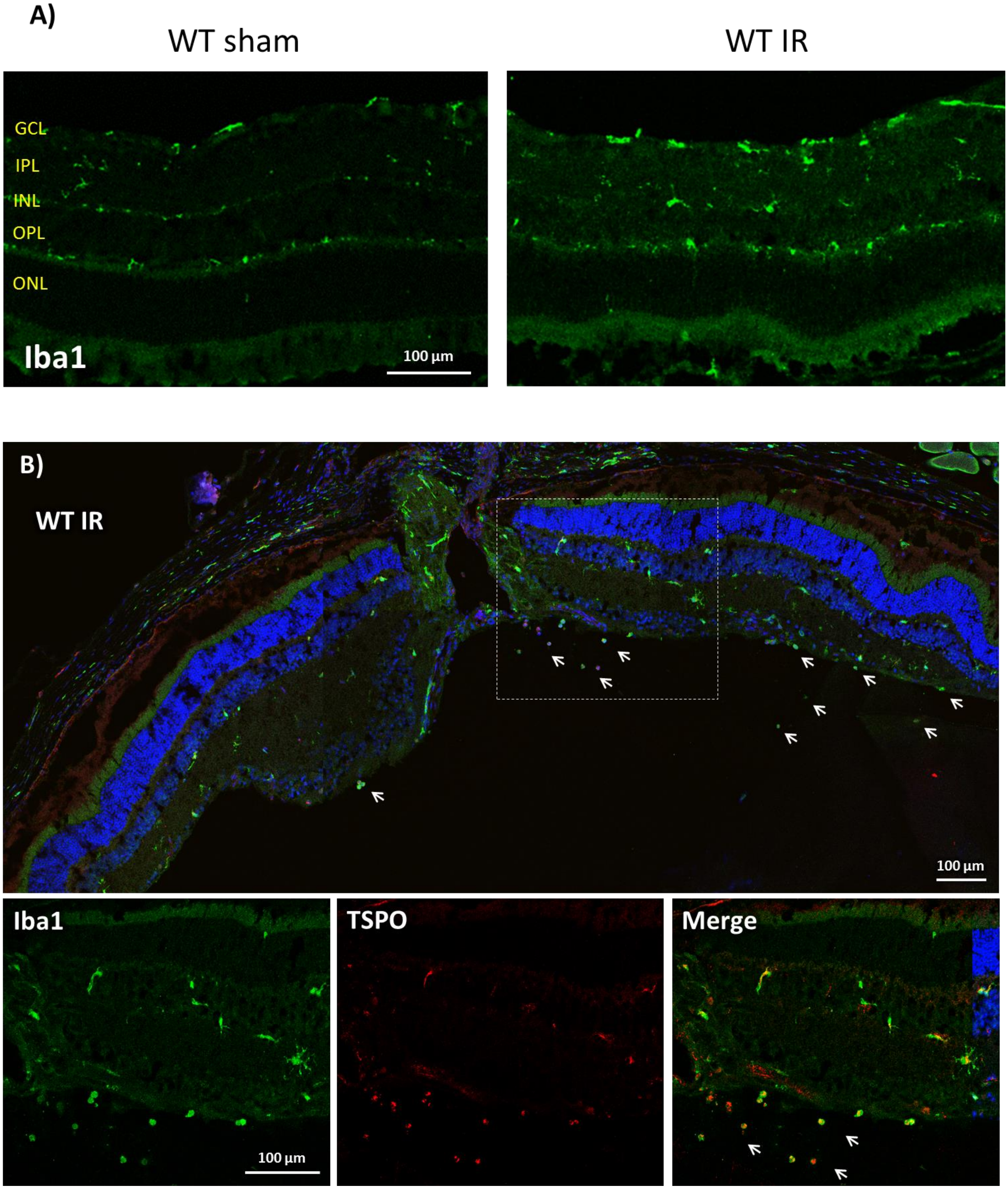

A)
WT sham
WT IR
GCL
IPL
INL
OPL
ONL
Iba1
100 µm
B)
WT IR
100 µm
Iba1
TSPO
Merge
100 µm

Fig S3. Increased myeloid cell activation/infiltration after IR injury. A) Staining on WT retina cross sections 5 days after IR injury showed increased Iba1 positive cells. **B)** Staining WT eyeballs cross sections for Iba1 and TSPO (markers for macrophages/microglia) showed infiltrating myeloid cells into the vitreous at 48 h after IR injury, Iba1 (green), TSPO (red), and DAPI (blue), scale bar = 100 μm.

S4

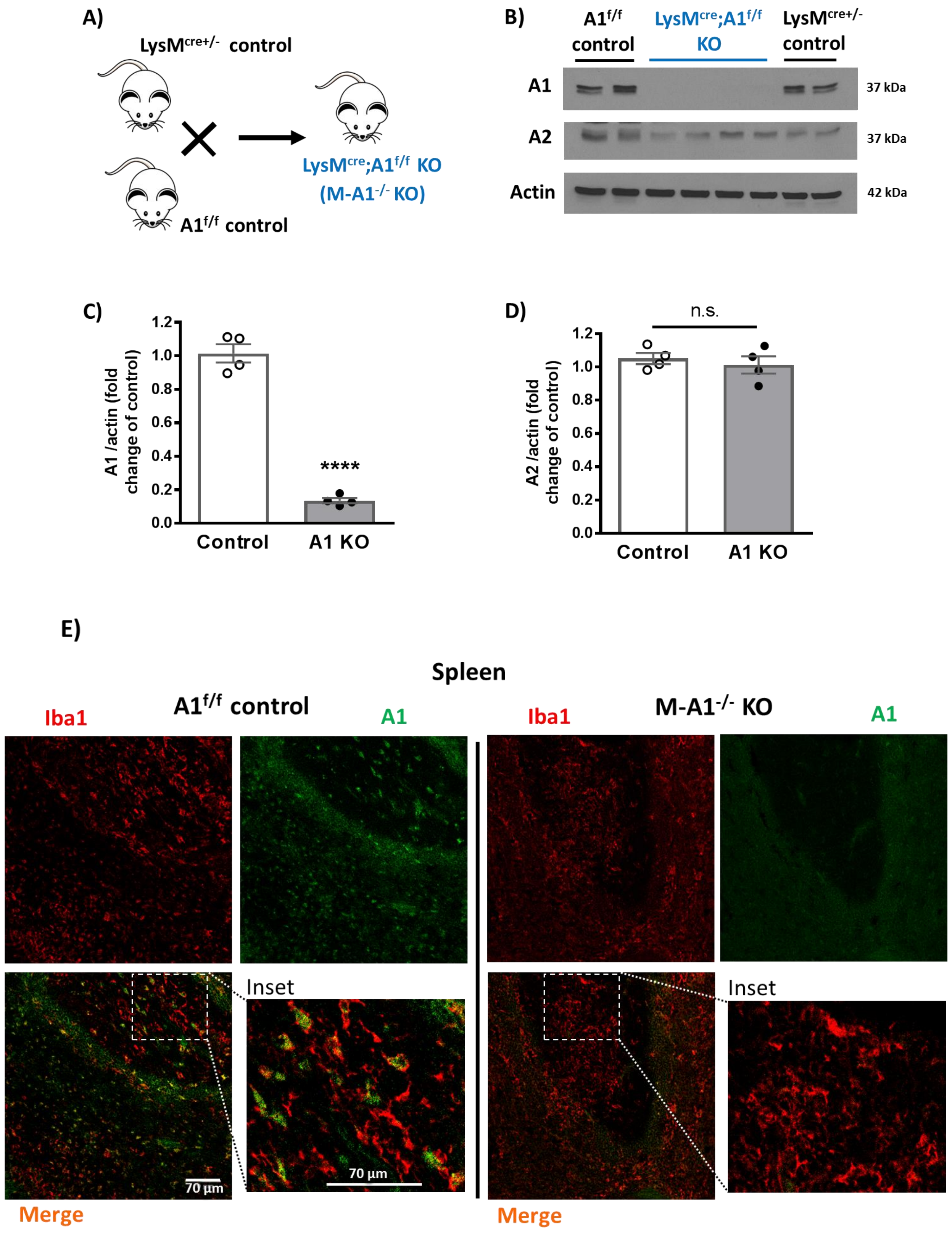

A)
LysMcre+/- control
A1f/f control
LysMcre;A1f/f KO
(M-A1-/- KO)
B)
A1f/f control
LysMcre;A1f/f KO
LysMcre+/- control
A1
A2
Actin
37 kDa
37 kDa
42 kDa
C)
A1 /actin (fold change of control)
1.2
1.0
0.8
0.6
0.4
0.2
0.0
****
Control
A1 KO
D)
n.s.
A2 /actin (fold change of control)
1.2
1.0
0.8
0.6
0.4
0.2
0.0
Control
A1 KO
E)
Spleen
A1f/f control
Iba1
A1
M-A1-/- KO
Iba1
A1
Inset
70 µm
70 µm
Merge
Inset
Merge

Fig S4. Confirmation of myeloid A1 deletion. A) Diagram represents crossing of floxed A1 mouse ($A1^{f/f}$) with mouse expressing Cre under LysM promoter ($LysM^{Cre+/-}$) to generate myeloid specific A1 KO mice ($M\text{-}A1^{-/-}$), **B)** Peritoneal macrophages were collected from $M\text{-}A1^{-/-}$ mice and their controls ($A1^{f/f}$ and $LysM^{Cre+/-}$) through peritoneal lavage with PBS, and cell lysates were run SDS-PAGE (each lane represents pooled macrophages from 2 or more mice). Western blotting showed no detectable band of A1 in $M\text{-}A1^{-/-}$ macrophages compared to controls. **C)** & **D)** Densitometric analyses of western blot band showed significant decrease in A1 protein by 90% in $M\text{-}A1^{-/-}$ macrophages while A2 expression showed no difference between groups under basal conditions (no stimulation). The two control groups were not different and therefore were pooled together for analyses, ****$p<0.0001$. **E)** Cross-sectional staining of spleen specimens from loxP control ($A1^{f/f}$) and $M\text{-}A1^{-/-}$ mice showed co-localization of A1 (green) with the macrophage marker Iba1 (red) in $A1^{f/f}$ but not in $M\text{-}A1^{-/-}$, scale bar = 70 µm.

S5

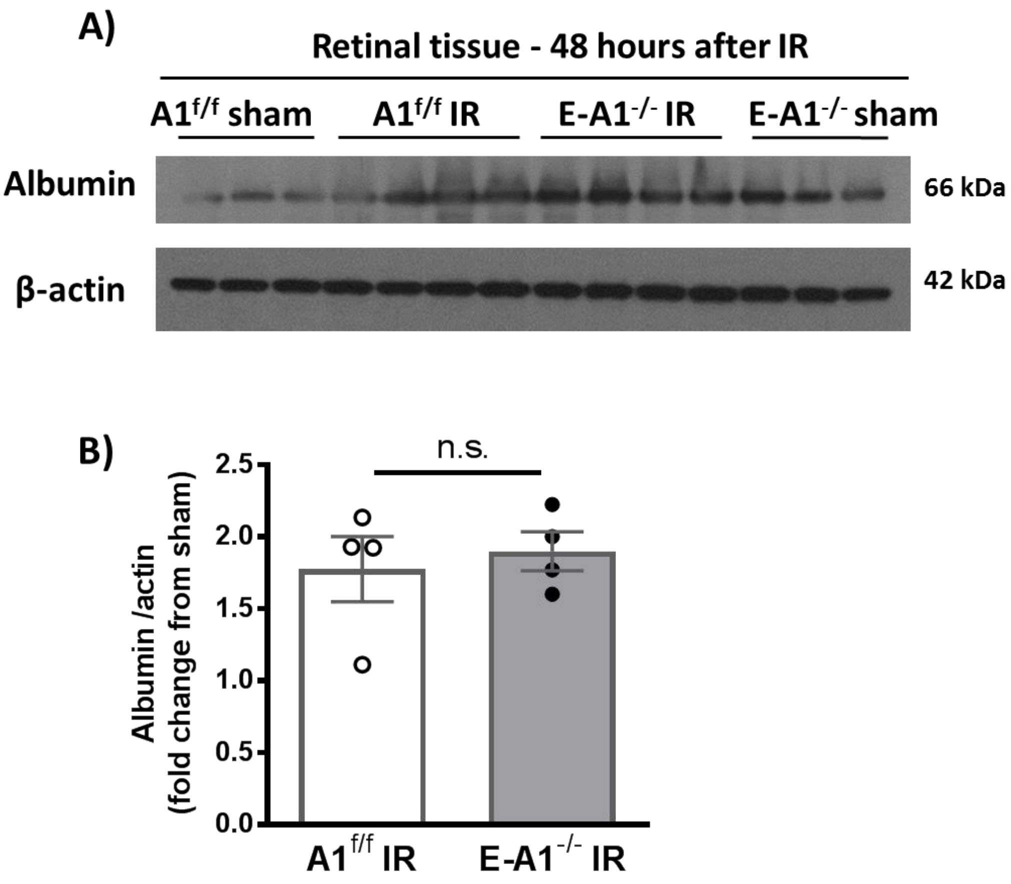

Fig S5. Endothelial specific A1 deletion does not affect blood-retinal barrier permeability after IR injury. A) Western blotting conducted on retinas collected from transcardially PBS-perfused mice at 48 h after IR injured showed increased albumin extravasation compared to sham retinas. Endothelial specific A1 KO mice did not show any difference in albumin extravasation as compared to WT retinas. **B)** Quantification of Western blot albumin band.

S6

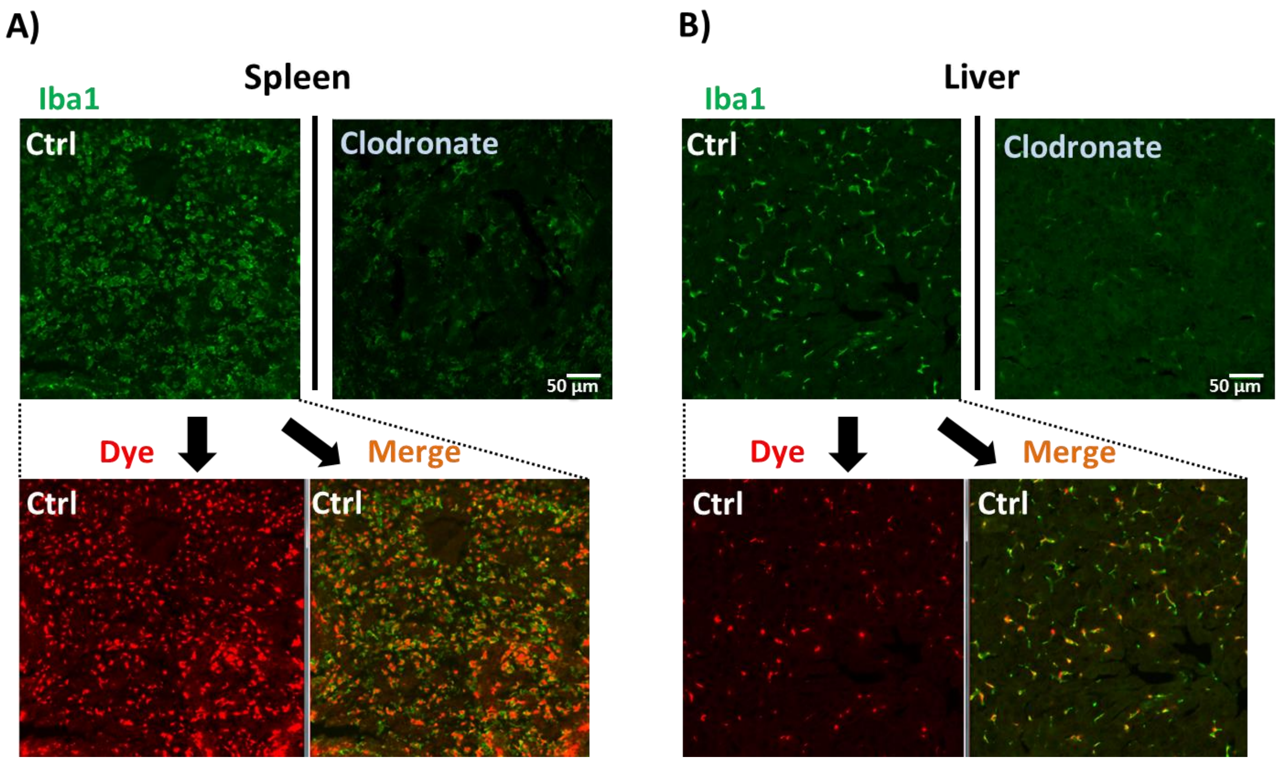

Fig S6. Confirmation of systemic monocytes/macrophages depletion with clodronate liposomes. Cross-sectional staining of spleen **(A)** and liver **(B)** specimens collected 2 days after intraperitoneal injection of clodronate or control red fluorescent liposomes. The macrophage marker Iba1 (green) showed >80% macrophage depletion in both organs after clodronate liposomes injection. Specimens from mice injected with control liposomes showed macrophages displaying red fluorescence confirming the uptake of liposomes without induction of apoptosis. Scale bar = 50 µm.

S7

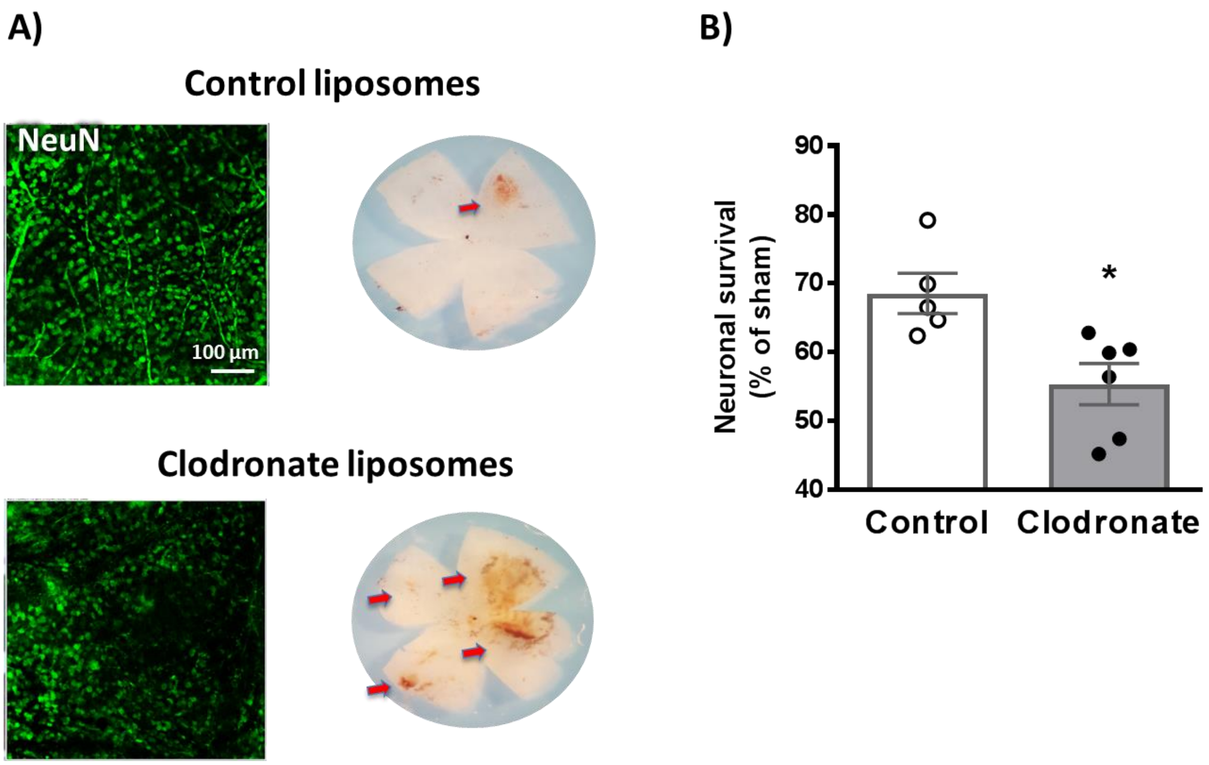

Fig. S7: Systemic monocytes/macrophage depletion worsens IR injury-induced neurodegeneration. A) Depletion of monocytes/macrophages in WT mice using clodronate liposomes resulted in increased neuronal loss at 7 days as well as increased bleeding as shown in images of dissected retina flat-mounts (red arrows). **B)** Quantification of NeuN positive cells, n=5 for control and 6 for clodronate liposomes, *p<0.05.

S8

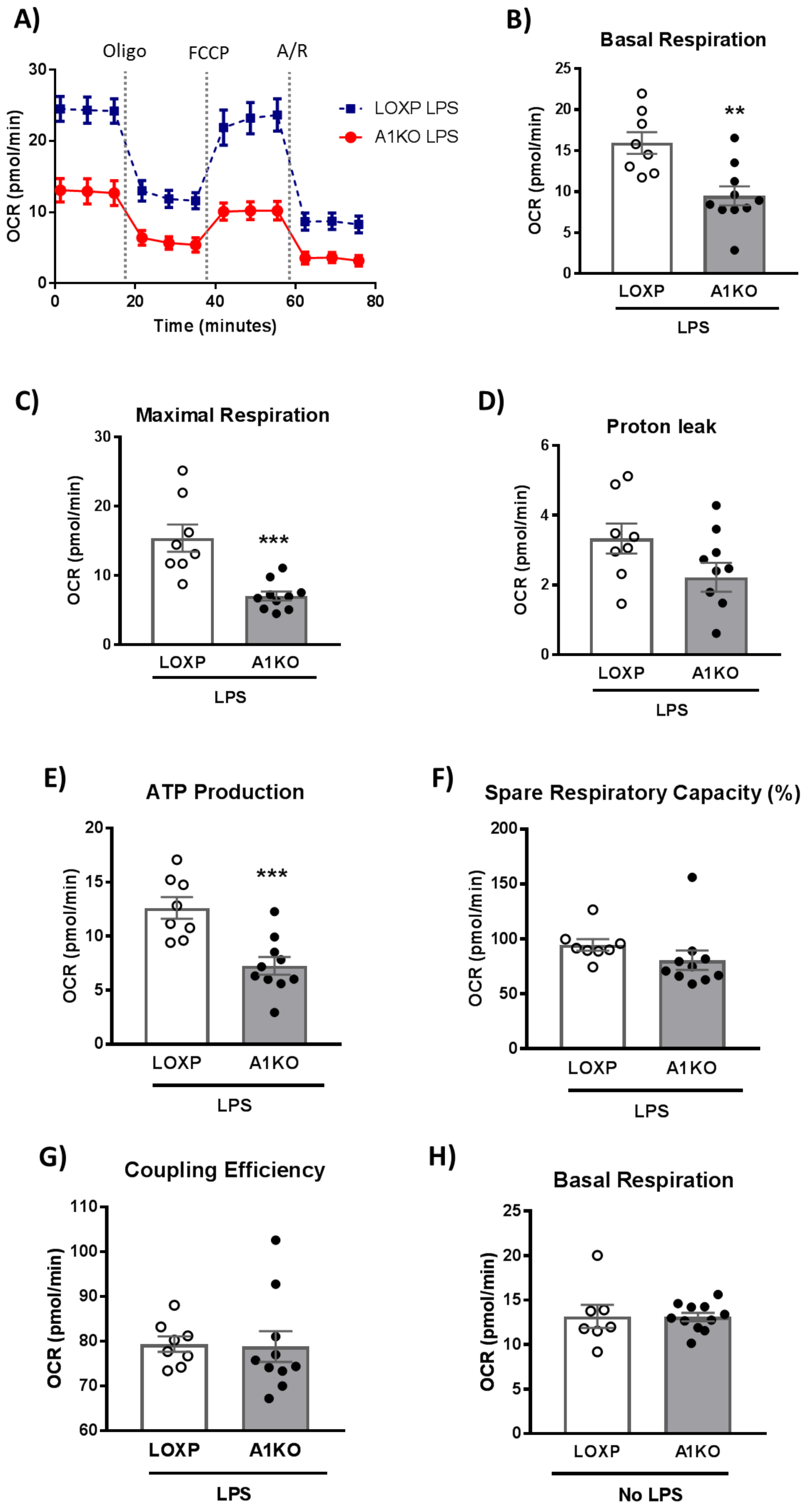
A)
Oligo
FCCP
A/R
LOXP LPS
A1KO LPS
OCR (pmol/min)
0
10
20
30
0
20
40
60
80
Time (minutes)
B)
Basal Respiration
OCR (pmol/min)
**
LOXP
A1KO
LPS
C)
Maximal Respiration
OCR (pmol/min)
***
LOXP
A1KO
LPS
D)
Proton leak
OCR (pmol/min)
LOXP
A1KO
LPS
E)
ATP Production
OCR (pmol/min)
***
LOXP
A1KO
LPS
F)
Spare Respiratory Capacity (%)
OCR (pmol/min)
LOXP
A1KO
LPS
G)
Coupling Efficiency
OCR (pmol/min)
LOXP
A1KO
LPS
H)
Basal Respiration
OCR (pmol/min)
LOXP
A1KO
No LPS

Fig. S8: A1 deletion augments LPS-induced mitochondrial dysfunction. A) Mito stress test showing change in A1 KO and control BMDMs OCR with time after stimulation with LPS (100 ng/ml) for 24h. **B** to **G)** Mitochondrial respiration parameters were decreased in A1 KO macrophages as compared to control after LPS treatment, n=8 per group, *p<0.05, **p<0.01, ***p<0.001. **H)** There was no change in basal respiration between untreated A1 KO and control macrophages.

S9

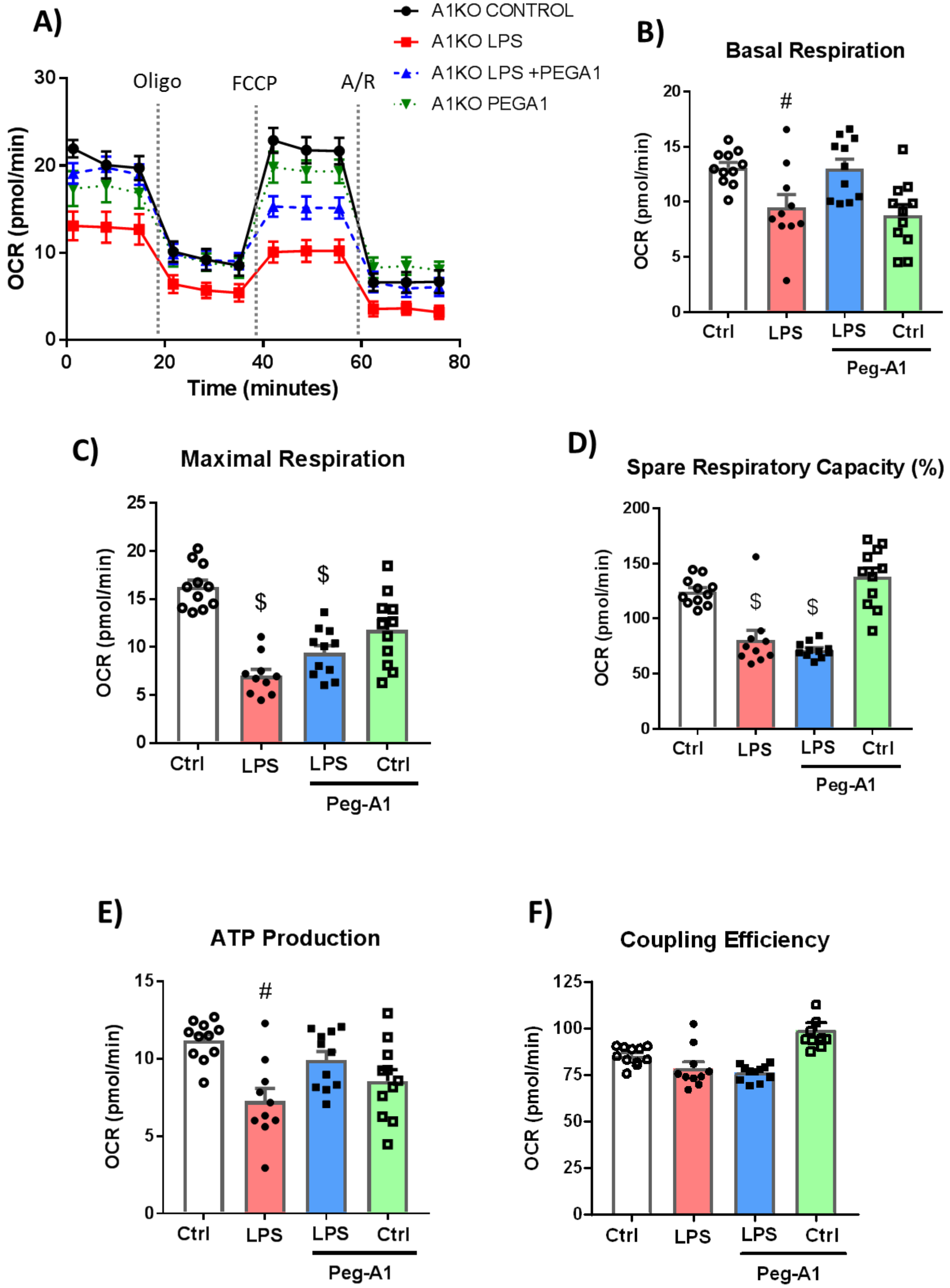
A)
A1KO CONTROL
A1KO LPS
A1KO LPS +PEGA1
A1KO PEGA1
Oligo
FCCP
A/R
OCR (pmol/min)
Time (minutes)
B)
Basal Respiration
C)
Maximal Respiration
D)
Spare Respiratory Capacity (%)
E)
ATP Production
F)
Coupling Efficiency
Ctrl
LPS
LPS
Ctrl
Peg-A1
#
$

Fig. S9: PEG-A1 treatment protects against LPS-induced mitochondrial dysfunction in A1 KO BMDMs. A) Mito stress test showing change in A1 KO BMDMs OCR with time after stimulation with LPS (100 ng/ml) for 24h ± PEG-A1 (1 μg/ml). **B** to **F)** Mitochondrial respiration parameters were decreased with LPS treatment. PEG-A1 significantly rescued this decrease. $^{\$}$p<0.01 vs controls, $^{\#}$p<0.01 vs respective control, and LPS+PEG-A1, n=10 per group.

S10

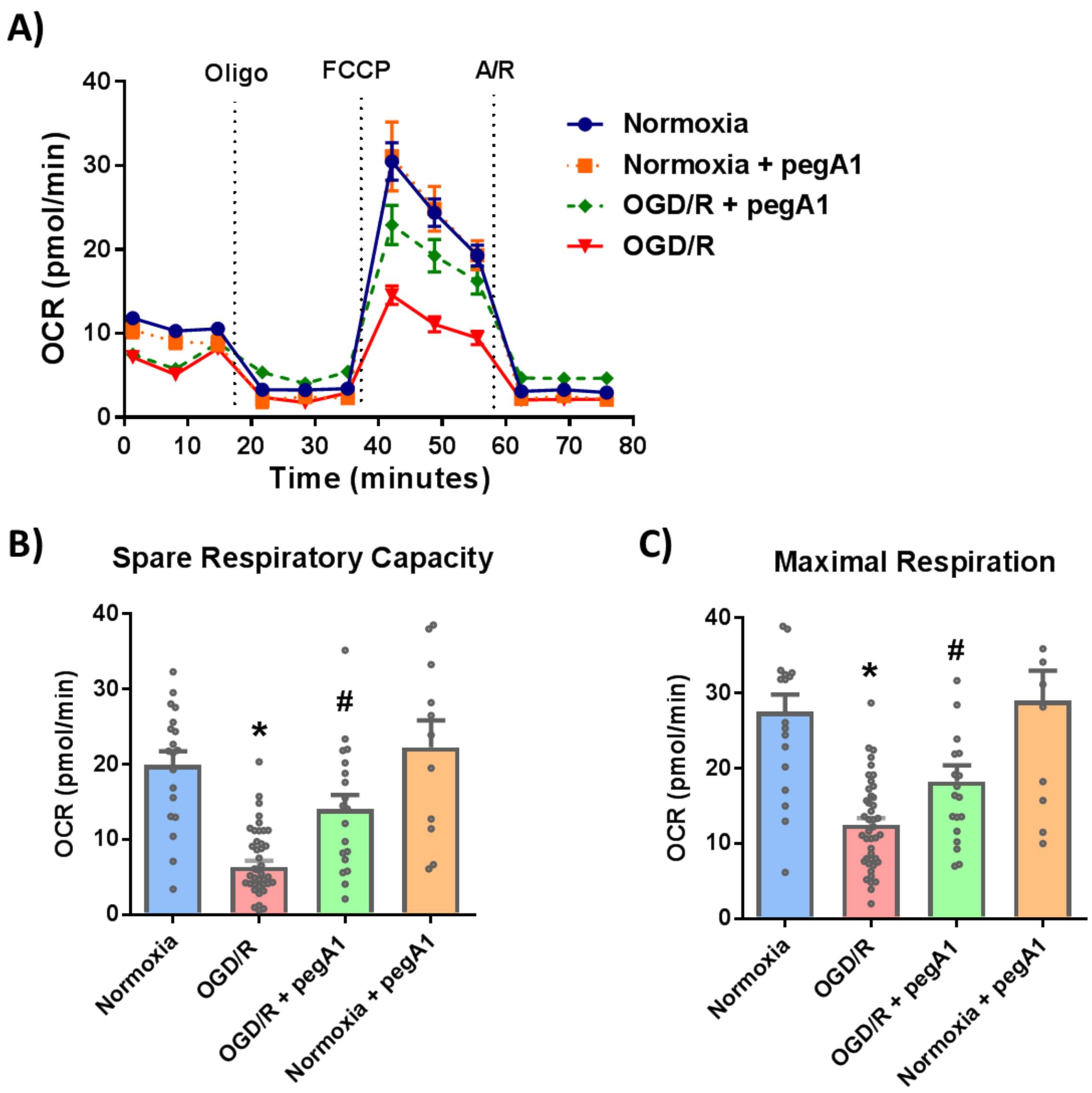

Fig. S10: PEG-A1 enhances mitochondrial bioenergetics in endothelial cells after OGD/R. A) Bovine retinal endothelial cells (BRECs) subjected to OGD for 5 h and 1 h reoxygenation (R) showed reduced mitochondrial respiration as measured by the oxygen consumption rate (OCR) using Seahorse XFe96. **B, C)** PEG-A1 treatment (1 μg/ml) improved maximal respiration and spare respiratory capacity, n=10 per group, *p<0.05 vs Normoxia, #p<0.05 vs OGD/R.

S11

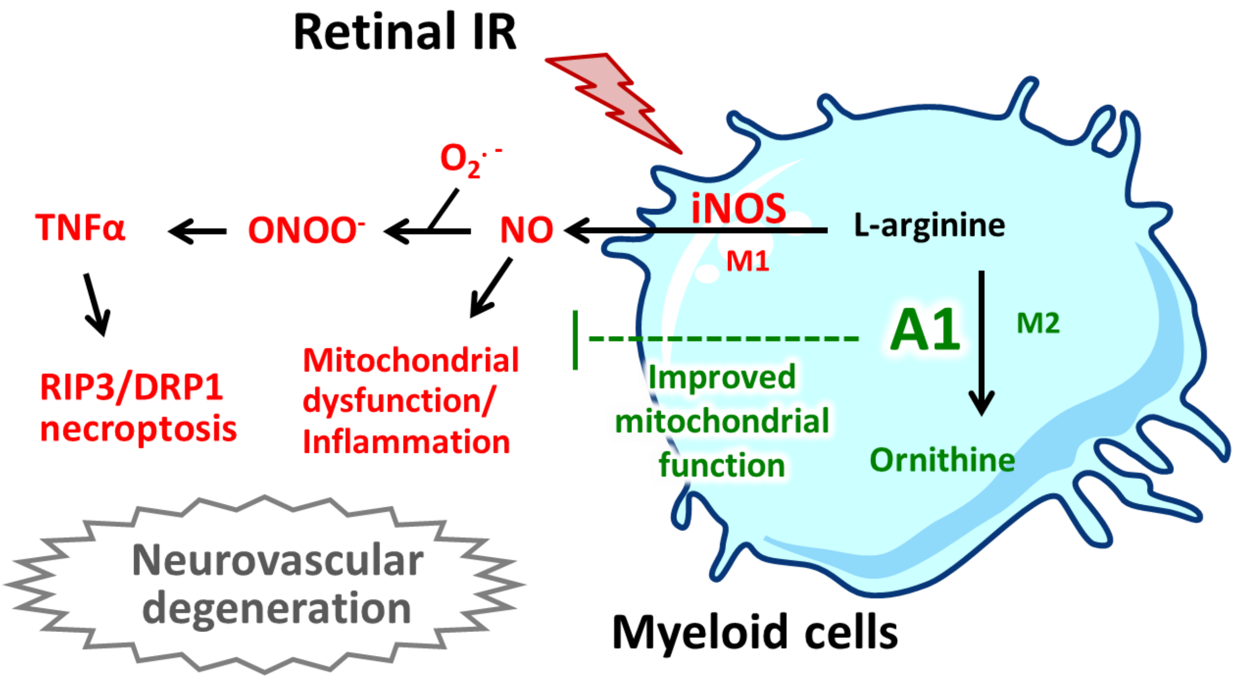

Fig. S11: Schematic representation of the suggested mechanism of A1-mediated dampening of macrophage inflammatory response after retinal IR. Retinal IR injury induces iNOS expression in macrophages leading to increased NO production. NO subsequently leads to decreased mitochondrial oxidative phosphorylation via nitrosylation of electron transport chain. NO can combine with superoxide anion ($O_2^{\cdot-}$) to form peroxynitrite ($ONOO^-$) which induces TNFα expression leading to RIP3/DRP1 mediated neurovascular degeneration and necroptosis. A1 reduces iNOS mediated NO formation through depletion of the substrate L-arginine. Thus, A1 restores mitochondrial oxidative phosphorylation and decreases macrophage inflammatory response. This is associated with decreased neurovascular degeneration after retinal IR. The figure was produced using Servier Medical Art (http://smart.servier.com/)

REFERENCES:

1. Rosenbaum DM, Degterev A, David J, Rosenbaum PS, Roth S, Grotta JC*, et al.* Necroptosis, a novel form of caspase-independent cell death, contributes to neuronal damage in a retinal ischemia-reperfusion injury model. *J Neurosci Res* 2010, **88**(7)**:** 1569-1576.

2. Shosha E, Xu Z, Yokota H, Saul A, Rojas M, Caldwell RW*, et al.* Arginase 2 promotes neurovascular degeneration during ischemia/reperfusion injury. *Cell Death Dis* 2016, **7**(11)**:** e2483.

3. Bhatta A, Yao L, Xu Z, Toque HA, Chen J, Atawia RT*, et al.* Obesity-induced vascular dysfunction and arterial stiffening requires endothelial cell arginase 1. *Cardiovascular research* 2017, **113**(13)**:** 1664-1676.

4. Shosha E, Xu Z, Narayanan SP, Lemtalsi T, Fouda AY, Rojas M*, et al.* Mechanisms of Diabetes-Induced Endothelial Cell Senescence: Role of Arginase 1. *International journal of molecular sciences* 2018, **19**(4).
